# Machine learning-based analysis of risk factors for atrial fibrillation recurrence after Cox-Maze IV procedure in patients with atrial fibrillation and chronic valvular disease: A retrospective cohort study with a control group

**DOI:** 10.3389/fcvm.2023.1140670

**Published:** 2023-03-24

**Authors:** Zenan Jiang, Long Song, Chunshui Liang, Hao Zhang, Haoyu Tan, Yaqin Sun, Ruikang Guo, Liming Liu

**Affiliations:** ^1^ Department of Cardiovascular Surgery, the Second Xiangya Hospital of Central South University, Changsha, China; ^2^ Department of Cardiovascular Surgery, Xinqiao Hospital, Army Medical University, Chongqing, China

**Keywords:** Cox-Maze IV procedure, atrial fibrillation, machine learning, recurrence prediction, risk factors, feature importance analysis

## Abstract

**Objectives:**

To evaluate the efficacy of the Cox-Maze IV procedure (CMP-IV) in combination with valve surgery in patients with both atrial fibrillation (AF) and valvular disease and use machine learning algorithms to identify potential risk factors of AF recurrence.

**Methods:**

A total of 1,026 patients with AF and valvular disease from two hospitals were included in the study. 555 patients received the CMP-IV procedure in addition to valve surgery and left atrial appendage ligation (CMP-IV group), while 471 patients only received valve surgery and left atrial appendage ligation (Non-CMP-IV group). Kaplan–Meier analysis was used to calculate the sinus rhythm maintenance rate. 58 variables were selected as variables for each group and 10 machine learning models were developed respectively. The performance of the models was evaluated using five-fold cross-validation and metrics including F1 score, accuracy, precision, and recall. The four best-performing models for each group were selected for further analysis, including feature importance evaluation and SHAP analysis.

**Results:**

The 5-year sinus rhythm maintenance rate in the CMP-IV group was 82.13% (95% CI: 78.51%, 85.93%), while in the Non-CMP-IV group, it was 13.40% (95% CI: 10.44%, 17.20%). The eXtreme Gradient Boosting (XGBoost), LightGBM, Category Boosting (CatBoost) and Random Fores (RF) models performed the best in the CMP-IV group, with area under the curve (AUC) values of 0.768 (95% CI: 0.742, 0.786), 0.766 (95% CI: 0.744, 0.792), 0.762 (95% CI: 0.723, 0.801), and 0.732 (95% CI: 0.701, 0.763), respectively. In the Non-CMP-IV group, the LightGBM, XGBoost, CatBoost and RF models performed the best, with AUC values of 0.738 (95% CI: 0.699, 0.777), 0.732 (95% CI: 0.694, 0.770), 0.724 (95% CI: 0.668, 0.789), and 0.716 (95% CI: 0.656, 0.774), respectively. Analysis of feature importance and SHAP revealed that duration of AF, preoperative left ventricular ejection fraction, postoperative heart rhythm, preoperative neutrophil-lymphocyte ratio, preoperative left atrial diameter and heart rate were significant factors in AF recurrence.

**Conclusion:**

CMP-IV is effective in treating AF and multiple machine learning models were successfully developed, and several risk factors were identified for AF recurrence, which may aid clinical decision-making and optimize the individual surgical management of AF.

## 1. Introduction

Atrial fibrillation (AF) is a common clinical tachyarrhythmia and is the most common arrhythmia in adult cardiac surgery patients, representing about one-third of all arrhythmias with a global incidence of nearly 1% (1, 2). It increases the risk of ischemic stroke (3, 4), myocardial infarction (5), and renal insufficiency (6).

Cox-Maze Procedure (CMP) is currently considered the most effective surgical treatment for AF and is the gold standard for surgical treatment of AF (7, 8). Cox-Maze IV Procedure (CMP-IV) replaces the “cut-and-sew” technique of the original CMP with lines of ablation created using bipolar radiofrequency energy (9–11). Some risk factors for the recurrence of AF after CMP have been identified, including enlarged left atrial diameter (LAD), failure to isolate the entire posterior left atrium, longer valvular disease duration, coronary artery disease, and larger right atrial diameter (12–15). However, there is still a lack of large-scale, long-term controlled studies, and the risk factors for AF recurrence after CMP-IV need further verification and evaluation. If further risk factors that influence the efficacy of CMP-IV can be identified, it may be possible to enhance the effectiveness of CMP-IV in treating AF.

Previous clinical studies have mainly used traditional statistical analysis methods such as linear and logistic regression, linear discriminant analysis, and correlation analysis to investigate relationships between variables and analyze clinical data (14, 15). However, these methods have limitations in terms of their ability to handle large amounts of data and explore nonlinear relationships (16, 17). Machine learning is a branch of artificial intelligence, which can learn from data and extract features for tasks such as classification, prediction, and clustering (18). Currently, machine learning is increasingly integrated into clinical practice, with applications ranging from preclinical data processing to patient stratification and treatment decision-making, mainly including disease diagnosis, treatment risk assessment, drug production and medical data analysis (19–22). The purpose of this study is to evaluate the efficacy of CMP-IV in patients with chronic valvular disease and AF through a retrospective cohort control study and to construct machine learning models to evaluate the risk factors for the recurrence of AF after CMP-IV.

## 2. Materials and methods

This clinical study has been registered on the Chinese clinical trial website (ChiCTR1900023775). This research was funded by the National Key Research and Development Program of China (2018YFC1311204). The studies involving human participants were reviewed and approved by the Ethics Committee of the Second Xiangya Hospital of Central South University and Xinqiao Hospital Affiliated to Army Medical University. Written consent was obtained from all patients before surgery.

### 2.1. Data source and study population

In this study, we enrolled a total of 1,026 eligible patients with AF and chronic valvular disease at the Second Xiangya Hospital of Central South University and Xinqiao Hospital Affiliated to Army Medical University between January 2012 and December 2019. Among them, 555 patients underwent CMP-IV with valve surgery and left atrial appendage ligation (CMP-IV group), as well as 471 patients only underwent valve surgery and left atrial appendage ligation without CMP-IV at the same hospitals during the same period (Non-CMP-IV group). The inclusion and exclusion criteria for the study are detailed in Supplementary Material.

### 2.2. Definition of AF recurrence and follow-up records

AF recurrence included the continuous occurrence of AF, atrial flutter and atrial tachycardia recorded on the electrocardiogram (ECG) for more than 30 s. Non-AF recurrent conditions include sinus rhythm, junctional rhythm, atrial premature beats, and ventricular premature beats. Diagnostic records: physical examination and auscultation, ECG, 24-h dynamic ECG, and related medical records. The postoperative follow-up period for included patients began at 6 months after surgery and continued for at least 12 months. Follow-up records were collected every 12 months. During follow-up visits, symptoms and signs were recorded, and routine examinations such as cardiac ultrasound and ECG were performed. In cases where patients were unable to come to the hospital for a follow-up visit due to personal reasons, they were contacted by phone and recommended to complete the examination at a local hospital and report the results to their follow-up recorder. If a patient experienced palpitations or suspected recurrent AF, an ECG should be performed at any time and the results reported to the follow-up recorder.

### 2.3. Study variables and data processing

58 features were selected as variables in each group, including demographics, medical history, laboratory test results, and clinically relevant variables. Data were collected from the electronic medical records of the patients and underwent data cleaning and preprocessing to ensure quality and consistency. Specifically, we checked for and imputed missing values using the median value for numerical features and the mode for categorical features. We also checked for outliers and transformed skewed features using log transformation. By performing these data processing steps, we ensured the quality and consistency of the data used in our analysis. Both centers in this study performed data collection, data quality control, and unified data entry and storage in accordance with a uniform standard to ensure the compatibility of data.

### 2.4. Machine learning model establishment and model evaluation

We developed ten common machine learning models for the two groups respectively, including Support Vector Machine (SVM) (23), Logistic Regression (LR) (24), eXtreme Gradient Boosting (XGBoost) (25), Random Fores (RF) (26), Category Boosting (CatBoost) (27), Adaptive Boosting (AdaBoost) (28), Bootstrapped aggregation (Bagging) (29), Gradient boosting decision trees (GBDT) (30), Light Gradient Boosting Machine (LightGBM) (31) and Multilayer perceptron (MLP) (32). The optimal hyperparameter combination in machine learning models was determined through grid search. To evaluate the performance of these models, we performed five-fold cross-validation on the training data and calculated evaluation metrics including F1 score, Accuracy, Recall, Precision, and area under the curve (AUC), along with 95% confidence intervals. These metrics allowed us to assess the overall performance of the models and determine their suitability for use in clinical decision-making. The F1 score is the harmonic mean of precision and recall and is a useful metric for balancing the trade-off between these two metrics. Accuracy measures the percentage of correct predictions. Recall measures the percentage of true positive predictions among all actual positive cases. Precision measures the percentage of true positive predictions among all positive predictions. The Receiver Operating Characteristic (ROC) curve for each model was plotted in CMP-IV group and Non-CMP-IV group, using a 7:3 split for training and testing, to more clearly visualize the trade-off between true positive rate and false positive rate and evaluate the AUC (33).

### 2.5. Feature importance and explainable risk factor analysis

Based on the evaluation results, we selected four best-performing machine learning models in both the CMP-IV group and the Non-CMP-IV group. Feature importance analysis was performed on the selected models to identify the influence of the features on the predictions. The importance of each feature was calculated based on the internal mechanisms of the models. We analyzed the top 20 features in the 4 models of the two groups and analyzed their feature importance. The features were ranked based on their importance and visualized using feature importance plots. Additionally, we conducted SHapley Additive exPlanations (SHAP) analysis to interpret the results of our machine learning models for the two groups. Using SHAP force plots, we were able to examine the contribution of each feature to the model's prediction for each individual sample.

### 2.6. Statistical analysis

We performed statistical analysis using Python 3.8, the scikit-learn (sklearn) library 0.23.2, IBM SPSS Statistics 27 and R 4.0.2 to perform statistical analysis. Normally distributed continuous data have been expressed as mean ± standard deviation. Skewed distributed continuous data have been described as medians with interquartile ranges and were logarithmically transformed when necessary. Comparisons between categorical data were performed with the chi-square test, while continuous variables were assessed by *t*-test (for normal distribution) or nonparametric tests (for skewed distribution). A difference was considered statistically significant if *p* ≤ 0.01.

## 3. Results

### 3.1. Baseline characteristics

Baseline, hospitalization and follow-up characteristics for the CMP-IV group and Non-CMP-IV group are shown in Table 1. In a total of 1,026 patients, AF recurred in 117 of 555 (21.08%) in the CMP-IV group (the detailed information of baseline characteristics is presented in Supplementary Table S1) and 402 of 471 (85.35%) in the Non-CMP-IV group (the detailed information of baseline characteristics is presented in Supplementary Table S2). The median follow-up time was 5 years. There was no significant difference in age, duration of AF, and follow-up time between the two groups.

**Table 1:** Baseline, hospitalization and follow-up characteristics of the patients.

*n*	Total (*n* = 1,026)	Non-CMP-IV group (*n* = 471)	CMP-IV group (*n* = 555)	*p*
Gender, *n* (%)				
Female	713 (69.493)	343 (72.824)	370 (66.667)	0.033
Male	313 (30.507)	128 (27.176)	185 (33.333)	
Age, years	57.704 ± 7.911	57.416 ± 7.838	57.948 ± 7.964	0.284
Duration of AF, years	3.700 (2.000, 5.000)	3.000 (2.000, 6.000)	3.800 (2.800, 5.000)	0.683
Height, cm	158.565 ± 7.714	158.204 ± 7.275	158.871 ± 8.056	0.164
Weight, kg	58.417 ± 9.959	58.031 ± 9.525	58.744 ± 10.302	0.253
SBP, mm Hg	113.627 ± 15.414	113.327 ± 14.478	113.881 ± 16.162	0.563
DBP, mm Hg	71.435 ± 12.110	71.130 ± 13.257	71.694 ± 11.037	0.464
BMI, kg/m^2^	23.181 ± 3.237	23.167 ± 3.362	23.193 ± 3.126	0.898
Preoperative heart rate, bpm	89.244 ± 20.936	87.100 ± 21.463	91.063 ± 20.302	0.002
Preoperative LAD, mm	52.639 ± 9.693	53.336 ± 10.894	52.048 ± 8.498	0.038
Preoperative RAD, mm	39.474 ± 7.725	40.098 ± 8.862	38.944 ± 6.562	0.020
Preoperative LVD, mm	50.449 ± 8.437	51.059 ± 8.758	49.930 ± 8.118	0.033
Preoperative RVD, mm	37.407 ± 6.726	38.008 ± 7.424	36.896 ± 6.024	0.009
Preoperative LVEF, %	61.671 ± 8.478	61.943 ± 8.888	61.441 ± 8.108	0.345
Preoperative hypertension, *n* (%)	88 (8.577)	38 (8.068)	50 (9.009)	0.592
Preoperative diabetes, *n* (%)	34 (3.314)	17 (3.609)	17 (3.063)	0.626
Preoperative CHD, *n* (%)	73 (7.115)	31 (6.582)	42 (7.568)	0.540
History of preoperative cerebral infarction, *n* (%)	57 (5.556)	23 (4.883)	34 (6.126)	0.386
Preoperative pulmonary hypertension, *n* (%)	267 (26.023)	118 (25.053)	149 (26.847)	0.514
Smoking or drinking, *n* (%)	43 (4.191)	19 (4.034)	24 (4.324)	0.817
Preoperative NYHA, *n* (%)				
I&II	65 (6.335)	33 (7.006)	32 (5.766)	0.515
III	813 (79.240)	366 (77.707)	447 (80.541)	
IV	148 (14.425)	72 (15.287)	76 (13.694)	
HAS-BLED score, *n* (%)				
0	736 (71.735)	346 (73.461)	390 (70.270)	0.549
1	252 (24.561)	106 (22.505)	146 (26.306)	
2	34 (3.314)	17 (3.609)	17 (3.063)	
4	4 (0.390)	2 (0.425)	2 (0.360)	
CHA_2_DS_2_-VASc score, *n* (%)				
0	262 (25.536)	120 (25.478)	142 (25.586)	0.928
1	619 (60.331)	285 (60.510)	334 (60.180)	
2	72 (7.018)	32 (6.794)	40 (7.207)	
3	66 (6.433)	31 (6.582)	35 (6.306)	
4	3 (0.292)	1 (0.212)	2 (0.360)	
5	4 (0.390)	2 (0.425)	2 (0.360)	
Euro Score II score	1.639 ± 0.942	1.650 ± 0.949	1.630 ± 0.936	0.734
Preoperative WBC, 10^9^/L	6.123 ± 1.921	5.936 ± 1.876	6.281 ± 1.944	0.004
Preoperative NEUT%, %	59.507 ± 10.246	58.595 ± 10.279	60.280 ± 10.153	0.009
Preoperative RBC, 10^12^/L	4.768 ± 1.388	4.783 ± 1.451	4.754 ± 1.332	0.737
Preoperative hemoglobin, g/L	128.638 ± 15.768	127.444 ± 15.622	129.652 ± 15.821	0.025
Preoperative neutrophil, 10^9^/L	4.435 ± 1.739	4.430 ± 1.771	4.439 ± 1.710	0.934
Preoperative lymphocyte, 10^9^/L	1.866 ± 0.825	1.858 ± 0.833	1.874 ± 0.818	0.762
Preoperative NLR	2.351 (1.641, 3.510)	2.360 (1.604, 3.550)	2.342 (1.647, 3.419)	0.863
Preoperative PLT, 10^9^/L	189.235 ± 75.454	186.463 ± 76.597	191.587 ± 74.388	0.279
Preoperative INR	1.207 ± 0.940	1.293 ± 1.324	1.134 ± 0.363	0.012
Preoperative PT, s	13.118 ± 4.690	13.274 ± 5.124	12.985 ± 4.282	0.326
Preoperative AST, U/L	24.200 (16.500, 39.900)	22.300 (15.700, 38.100)	26.400 (17.300, 42.200)	0.001
Preoperative ALT, U/L	71.200 (58.100, 90.100)	69.800 (56.200, 88.000)	72.800 (60.200, 90.800)	0.034
Preoperative Creatinine, µmol/L	5.960 (4.380, 7.960)	5.940 (4.450, 7.800)	6.000 (4.330, 8.040)	0.851
Preoperative BUN, mmol/L	6.228 ± 2.566	6.213 ± 2.503	6.241 ± 2.618	0.863
Preoperative Tbil, μmol/L	15.200 (10.900, 21.600)	15.700 (11.300, 22.700)	14.700 (10.600, 21.000)	0.076
Type of surgery, *n* (%)				
AVR	14 (1.365)	8 (1.699)	6 (1.081)	0.009
DVR	386 (37.622)	157 (33.333)	229 (41.261)	
MVR	571 (55.653)	272 (57.749)	299 (53.874)	
TVP only	10 (0.975)	7 (1.486)	3 (0.541)	
MVP only	36 (3.509)	19 (4.034)	17 (3.063)	
TVR only	9 (0.877)	8 (1.699)	1 (0.180)	
MVP combined, *n* (%)	39 (3.801)	19 (4.034)	20 (3.604)	0.719
TVP combined, *n* (%)	752 (73.294)	273 (57.962)	479 (86.306)	<0.001
TVR combined, *n* (%)	31 (3.021)	27 (5.732)	4 (0.721)	<0.001
CBP time, min	103.297 ± 38.667	102.737 ± 43.122	103.773 ± 34.430	0.675
Aortic cross clamp time, min	65.228 ± 27.841	63.794 ± 31.113	66.445 ± 24.662	0.136
Left atrial thrombus, *n* (%)	119 (11.598)	67 (14.225)	52 (9.369)	0.015
Postoperative heart rhythm, *n* (%)				
Non-sinus rhythm	430 (41.910)	262 (55.626)	168 (30.270)	<0.001
Postoperative heart rate, bpm	84.209 ± 18.219	83.531 ± 21.436	84.784 ± 14.933	0.286
Postoperative LAD, mm	43.414 ± 8.249	44.128 ± 9.558	42.808 ± 6.889	0.013
Postoperative RAD, mm	36.161 ± 4.897	36.538 ± 5.046	35.841 ± 4.743	0.023
Postoperative LVD, mm	46.331 ± 5.922	47.128 ± 6.111	45.654 ± 5.670	<0.001
Postoperative RVD, mm	34.865 ± 4.533	34.984 ± 5.063	34.765 ± 4.027	0.449
Postoperative LVEF, %	64.769 ± 7.181	64.831 ± 7.248	64.716 ± 7.123	0.799
LOS in ICU, hours	39.443 ± 12.511	39.259 ± 12.436	39.600 ± 12.572	0.664
IABP or ECMO, *n* (%)	2 (0.195)	1 (0.212)	1 (0.180)	0.907
Cardioversion, *n* (%)	30 (2.924)	19 (4.034)	11 (1.982)	0.052
Permanent pacemaker, *n* (%)	7 (0.682)	6 (1.274)	1 (0.180)	0.034
Hemodialysis, *n* (%)	2 (0.195)	1 (0.212)	1 (0.180)	0.907
Postoperative AKI, *n* (%)	4 (0.390)	3 (0.637)	1 (0.180)	0.242
Postoperative cerebral infarction, *n* (%)	3 (0.292)	2 (0.425)	1 (0.180)	0.470
Length of hospital stay after surgery, days	11.000 (8.000, 15.000)	11.000 (8.000, 15.000)	11.000 (8.000, 16.000)	0.895
Follow-up time, years	5.000 (3.000, 6.000)	5.000 (4.000, 6.000)	5.000 (3.000, 6.000)	0.228
AF recurrence, *n* (%)	519 (50.585)	402 (85.350)	117 (21.081)	<0.001

AF, atrial fibrillation; LOS, length of stay; SBP, systolic blood pressure; DBP, diastolic blood pressure; BMI, body mass index; LAD, left atrial diameter; RAD, right atrial diameter; LVD, left ventricle diameter; RVD, right ventricle diameter; LVEF, left ventricular ejection fraction; CHD, coronary heart disease; PHTN, pulmonary hypertension; NLR, neutrophil-to-lymphocyte ratio; WBC, white blood cell count; NEUT%, neutrophil ratio; PLT, platelet; INR, international normalized ratio; PT, prothrombin time; AST, aspartate aminotransferase; ALT, alanine aminotransferase; Cr, creatinine; BUN, blood urea nitrogen; Tbil, total bilirubin; NYHA, New York Heart Association classification; AVR, aortic valve replacement; MVP, mitral valvuloplasty; TVP, tricuspid valvuloplasty; TVR, tricuspid valve replacement; CBP, cardiopulmonary bypass; AKI, acute kidney injury.

### 3.2. Sinus rhythm maintenance rate curve

**Figure 1:**
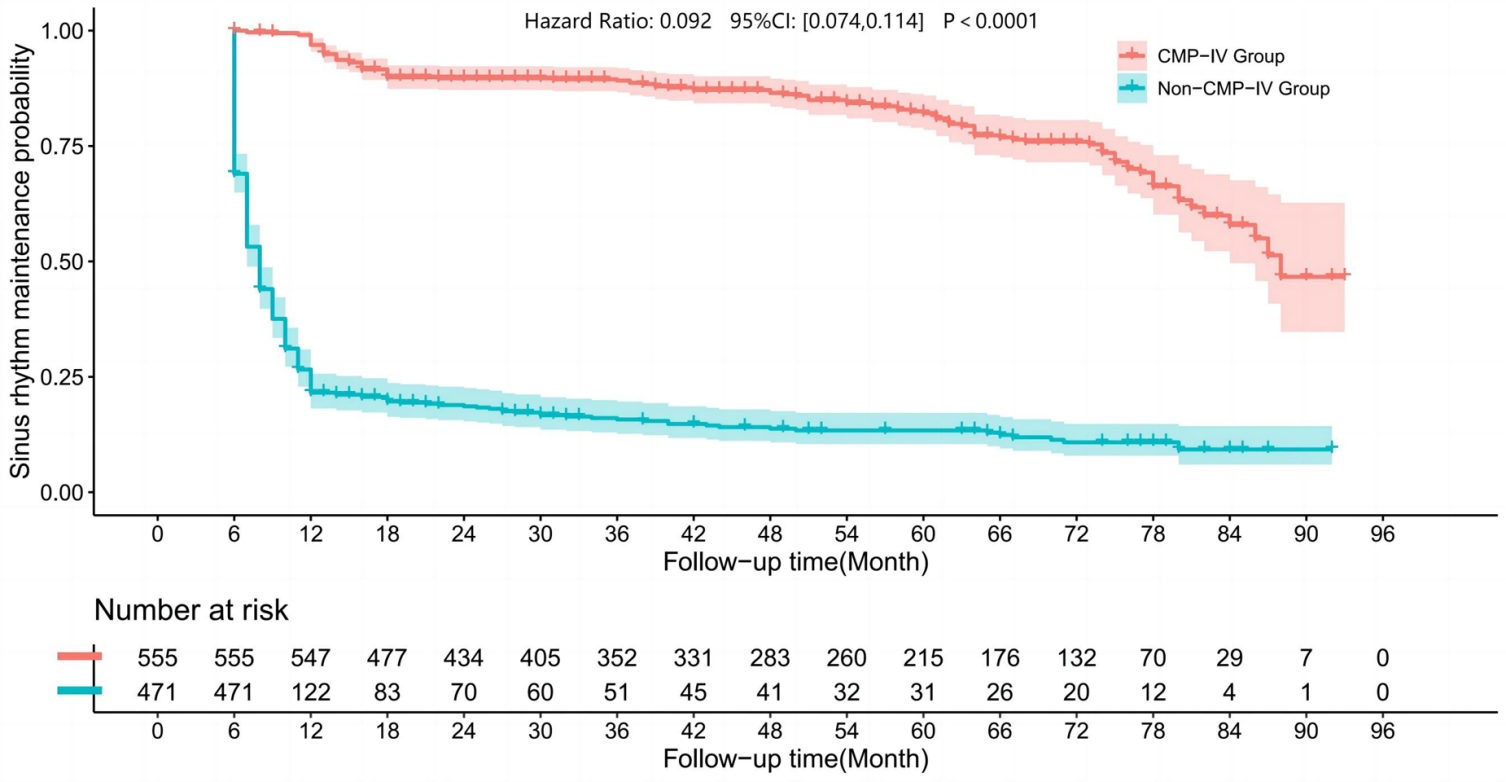
Kaplan–Meier curves were used to visualize the sinus rhythm maintenance rate in the two groups. The Log Rank rank sum test statistic for the comparison of survival time between the two groups was 673.932, indicating a significant difference in sinus rhythm maintenance rate between the CMP-IV group and the Non-CMP-IV group (*p* < 0.001). The median sinus rhythm maintenance time for the CMP-IV group was 88 months (95% CI: 84, NA) and for the Non-CMP-IV group was 8 months (95% CI: 7, 8). The hazard ratio for the Cox-Maze IV group compared to the Non-Cox-Maze IV group was 0.092 (95% CI: 0.074, 0.114). The follow-up time was plotted in months, and the number of patients at risk was shown below the graph.

We drew the sinus rhythm maintenance curves of the two groups, as shown in Figure 1. The results of our study showed that the sinus rhythm maintenance rate in the CMP-IV group was significantly higher than that in the Non-CMP-IV group. We used the Kaplan–Meier method to analyze the data and found that there was a statistically significant difference in the probability of recurrence between the two groups (*p* < 0.0001). The 5-year sinus rhythm maintenance rate in the CMP-IV group was 82.13% (95% CI: 78.51%, 85.93%), while in the Non-CMP-IV group, it was 13.40% (95% CI: 10.44%, 17.20%).

### 3.3. Development of postoperative AF recurrence prediction model

**Figure 2:**
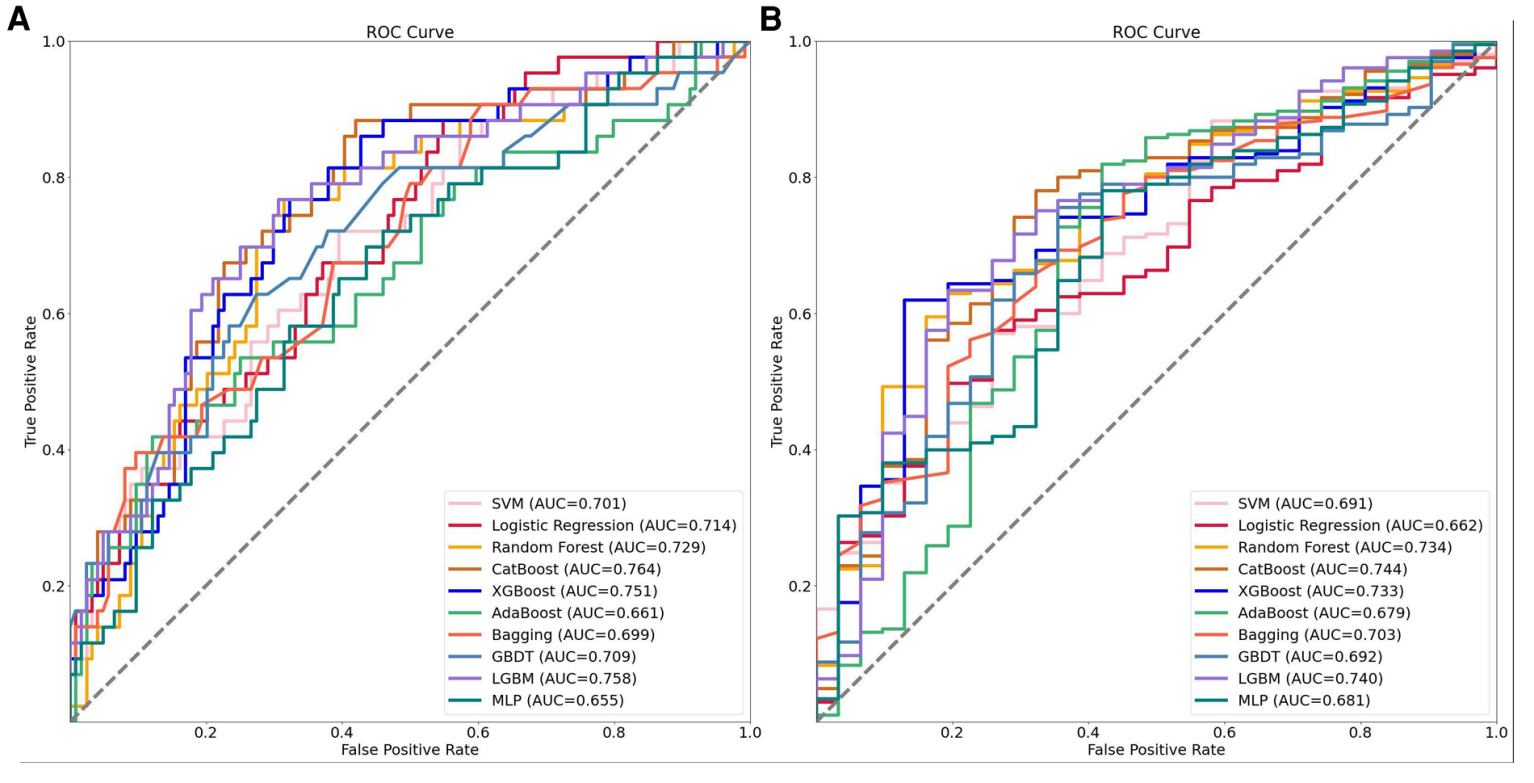
The receiver operating characteristic (ROC) curves of the 10 machine learning models established in the two groups. Panel **A** shows the ROC curves for the CMP-IV group, while Panel **B** shows the ROC curves for the No CMP-IV group. The acronyms in the legend stand for the following: SVM, support vector machine; Catboost, categorical boosting; XGBoost, extreme gradient boosting; AdaBoost, adaptive boosting; Bagging, bootstrapped aggregation; GBDT, gradient boosting decision trees; LightGBM, light gradient boosting machine; MLP, multilayer perceptron.

To evaluate the performance of the machine learning models, we conducted a five-fold cross-validation by dividing the data into training and test sets at a ratio of 8:2. The results of the model evaluation are shown in Table 2. In order to provide a more intuitive comparison, we also plotted the ROC curves of the models using data sets divided at a ratio of 7:3, as shown in Figure 2. Based on the ROC curves and the model evaluation results, we found that the CatBoost, LightGBM, XGBoost, and RF models in the CMP-IV group had the highest AUC of 0.768 (95% CI:0.742, 0.786), 0.766 (95% CI:0.744, 0.792), 0.762 (95% CI:0.723, 0.801), 0.732 (95% CI:0.701, 0.763), respectively. Among the 10 models in the Non-CMP-IV group, the LightGBM, XGBoost, RF, and CatBoost models performed the best (as shown in Table 3), with AUC of 0.714 (95% CI: 0.699, 0.813), 0.712 (95% CI: 0.669, 0.836), 0.711 (95% CI: 0.666, 0.849), and 0.699 (95% CI: 0.669, 0.831), respectively. Other model evaluation metrics including F1, ACC, Recall, and Precision are also at good performances in the 4 models of the two groups.

**Table 2:** Performance summary of machine learning models in CMP-IV group.

Model	Accuracy (95% CI)	Precision (95% CI)	Recall (95% CI)	F1 (95% CI)	AUC (95% CI)
XGBoost	0.802 (0.760, 0.844)	0.799 (0.782, 0.816)	0.633 (0.551, 0.772)	0.706 (0.664, 0.748)	0.768 (0.742, 0.786)
LGBM	0.804 (0.746, 0.836)	0.769 (0.796, 0.742)	0.582 (0.438, 0.732)	0.659 (0.575, 0.746)	0.766 (0.744, 0.792)
CatBoost	0.807 (0.729, 0.861)	0.764 (0.716, 0.812)	0.631 (0.532, 0.778)	0.697 (0.647, 0.747)	0.762 (0.723, 0.801)
RF	0.788 (0.769, 0.802)	0.736 (0.708, 0.764)	0.612 (0.598, 0.621)	0.671 (0.656, 0.687)	0.732 (0.701, 0.763)
GBDT	0.793 (0.785, 0.801)	0.717 (0.632, 0.802)	0.534 (0.503, 0.579)	0.611 (0.566, 0.655)	0.702 (0.665, 0.739)
Bagging	0.796 (0.744, 0.859)	0.716 (0.688, 0.744)	0.522 (0.387, 0.591)	0.615 (0.575, 0.656)	0.698 (0.652, 0.744)
LR	0.777 (0.732, 0.811)	0.689 (0.505, 0.819)	0.494 (0.405, 0.619)	0.588 (0.450, 0.702)	0.688 (0.664, 0.712)
SVM	0.795 (0.758, 0.836)	0.612 (0.568, 0.656)	0.512 (0.459, 0.565)	0.561 (0.512, 0.609)	0.687 (0.646, 0.728)
AdaBoost	0.773 (0.702, 0.812)	0.698 (0.601, 0.795)	0.533 (0.501, 0.565)	0.608 (0.552, 0.665)	0.668 (0.624, 0.712)
MLP	0.744 (0.688, 0.805)	0.637 (0.459, 0.781)	0.515 (0.488, 0.542)	0.575 (0.488, 0.656)	0.658 (0.616, 0.691)

XGBoost, extreme gradient boosting; LGBM, light gradient boosting machine; CatBoost, category boosting; RF, random forest; GBDT, Gradient boosting decision tree; Bagging, bootstrap aggregation; LR, logistic regression; SVM, support vector machine; AdaBoost, adaptive boosting; MLP, multi-layer perceptron; AUC, the area under the receiver operating characteristic curve.

**Table 3:** Performance summary of machine learning models in Non-CMP-IV group.

Model	Accuracy (95% CI)	Precision (95% CI)	Recall (95% CI)	F1 (95% CI)	AUC (95% CI)
LGBM	0.847 (0.830, 0.871)	0.857 (0.848, 0.877)	0.894 (0.889, 0.899)	0.895 (0.885, 0.908)	0.738 (0.699, 0.777)
XGBoost	0.832 (0.800, 0.861)	0.857 (0.843, 0.872)	0.888 (0.878, 0.898)	0.886 (0.867, 0.902)	0.732 (0.694, 0.770)
CatBoost	0.851 (0.840, 0.871)	0.856 (0.849, 0.870)	0.894 (0.888, 0.901)	0.897 (0.891, 0.908)	0.724 (0.668, 0.789)
RF	0.852 (0.843, 0.861)	0.853 (0.850, 0.861)	0.882 (0.866, 0.898)	0.898 (0.892, 0.903)	0.716 (0.656, 0.774)
GBDT	0.851 (0.843, 0.861)	0.853 (0.851, 0.858)	0.871 (0.846, 0.891)	0.898 (0.893, 0.903)	0.696 (0.647, 0.746)
Bagging	0.817 (0.779, 0.818)	0.859 (0.840, 0.865)	0.844 (0.815, 0.873)	0.876 (0.844, 0.883)	0.688 (0.622, 0.754)
SVM	0.843 (0.812, 0.861)	0.852 (0.845, 0.861)	0.868 (0.854, 0.882)	0.863 (0.844, 0.873)	0.672 (0.649, 0.740)
AdaBoost	0.807 (0.761, 0.830)	0.862 (0.838, 0.846)	0.855 (0.848, 0.862)	0.868 (0.842, 0.883)	0.682 (0.624, 0.742)
MLP	0.817 (0.796, 0.836)	0.849 (0.840, 0.858)	0.825 (0.766, 0.884)	0.874 (0.836, 0.893)	0.668 (0.634, 0.702)
LR	0.820 (0.800, 0.830)	0.851 (0.843, 0.864)	0.855 (0.839, 0.863)	0.878 (0.867, 0.884)	0.646 (0.608, 0.684)

LGBM, light gradient boosting machine; XGBoost, extreme gradient boosting; CatBoost, category boosting; RF, random forest; GBDT, Gradient boosting decision tree; Bagging, bootstrap aggregation; LR, logistic regression; SVM, support vector machine; AdaBoost, adaptive boosting; MLP, multi-layer perceptron; AUC, the area under the receiver operating characteristic curve.

### 3.4. Feature importance and model interpretability analysis

**Figure 3:**
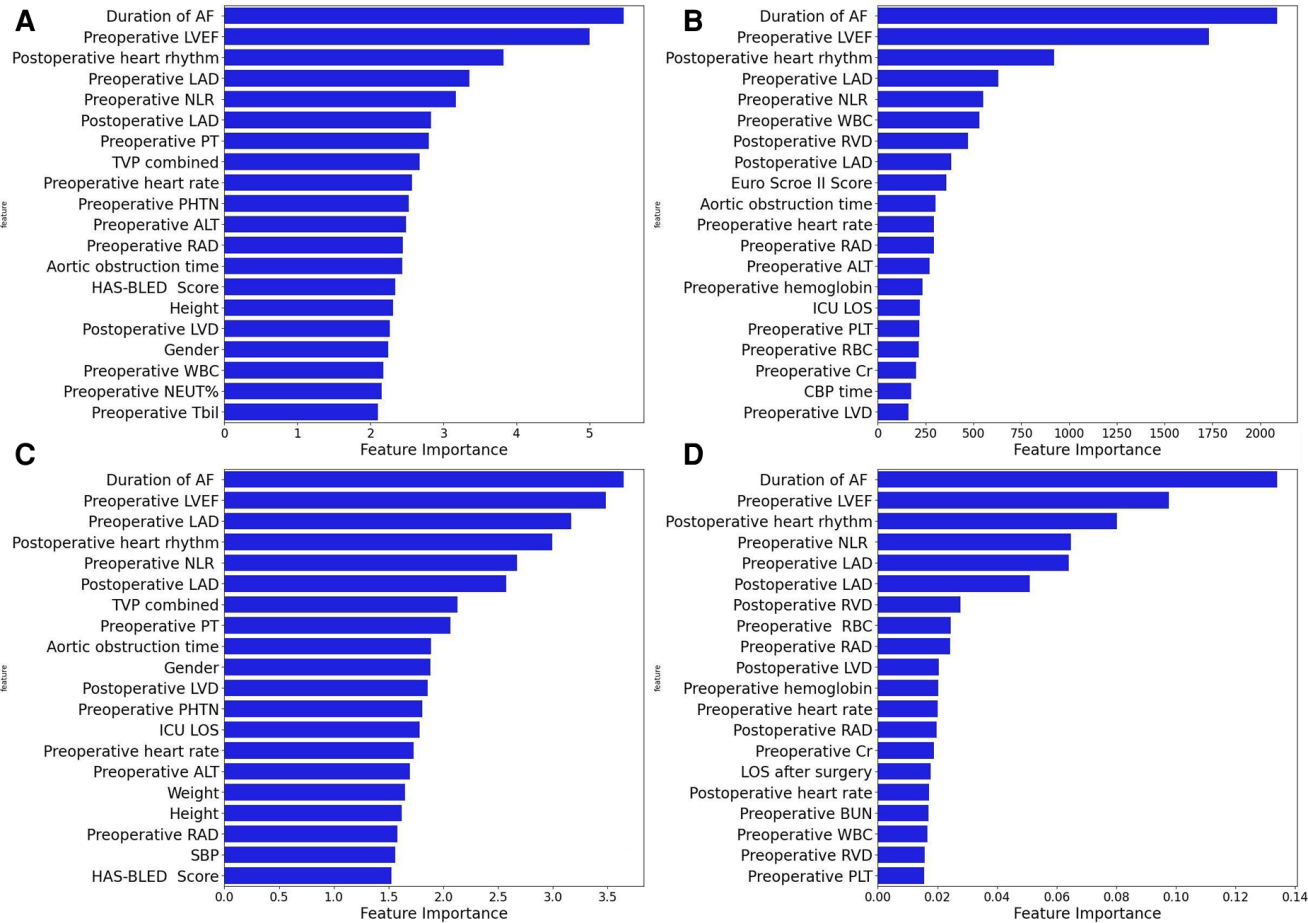
The feature importance plots of 4 machine learning models from the CMP-IV group are shown in **A–D**. Each plot is a bar graph with the feature importance on the x-axis and the names of each feature on the y-axis. The x-axis represents the feature importance, with the values indicating the contribution of the feature to the model. A higher value on the x-axis indicates a higher importance of the feature in the respective model. The models used are XGBoost (**A**), LightGBM (**B**), CatBoost (**C**), and Random Forest (**D**).

**Figure 4:**
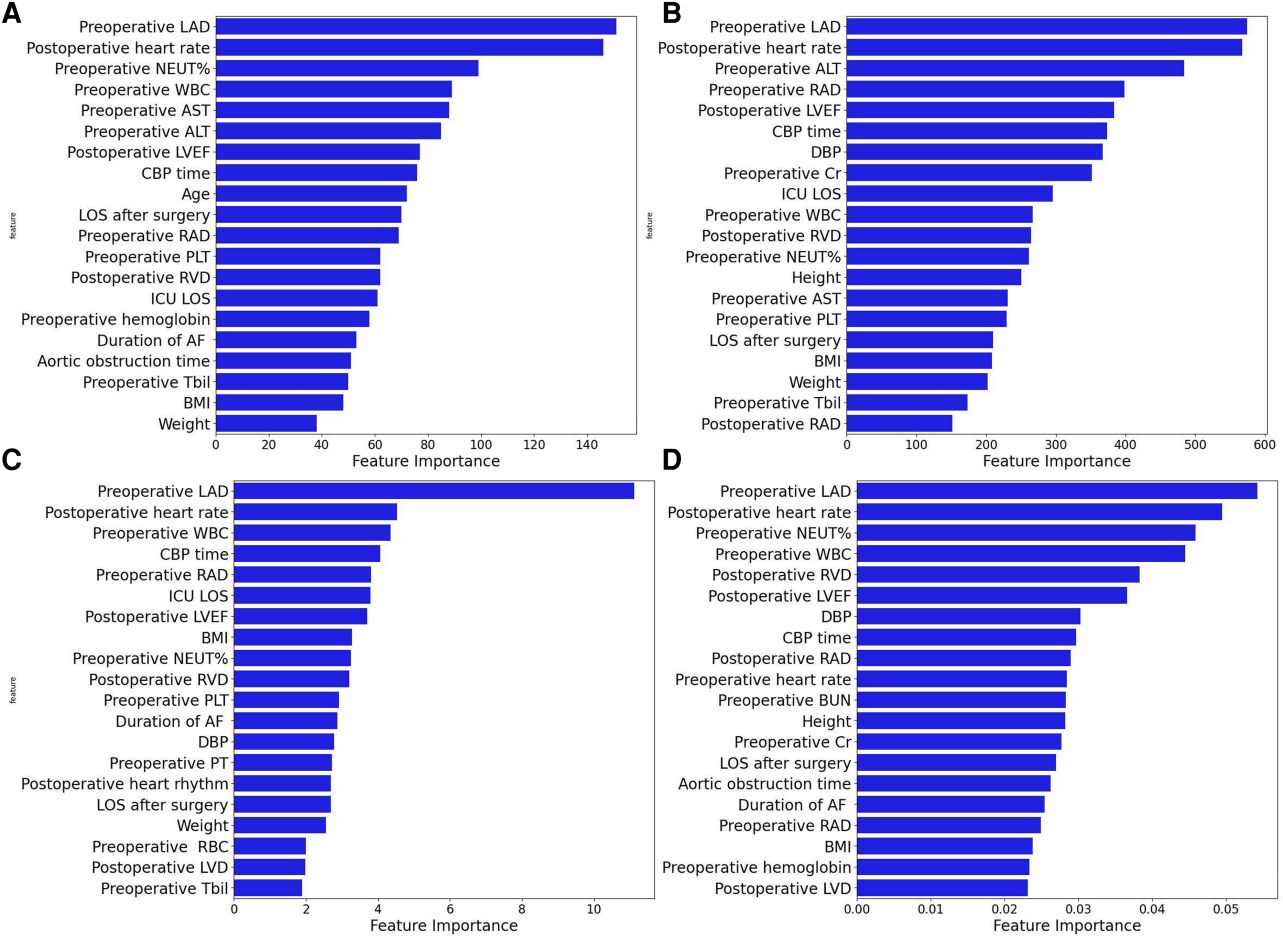
The feature importance plots of 4 machine learning models from the Non-CMP-IV group are shown in **A–D**. The models used are XGBoost (**A**), LightGBM (**B**), CatBoost (**C**), and Random Forest (**D**).

**Figure 5:**
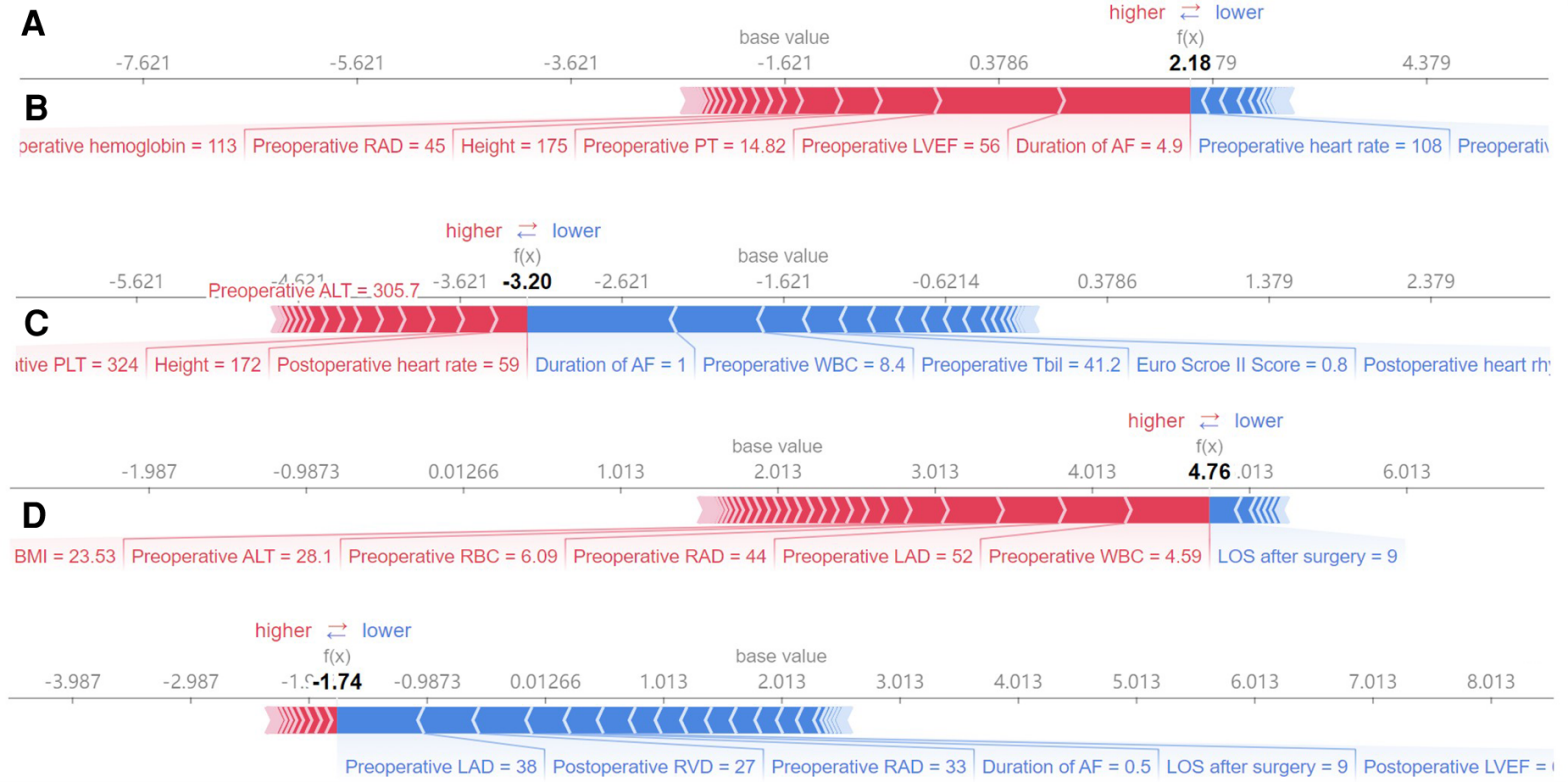
The SHAP force plots shown in **A–D** correspond to model-predicted risk scores for particular instances. The SHAP force plot is a graphical representation of the feature importance for a single sample in a machine learning model. The *x*-axis represents the different features, arranged from left to right. Each feature has a corresponding weight value, which indicates the impact of that feature on the prediction outcome. The size of the weight value indicates the magnitude of the impact of the feature on the prediction. The reference line represents the expected value of the prediction outcome if all feature values were set to their average value. The f(x) value represents the predicted outcome for the given sample. **A,B** show high and low risk examples for the CMP-IV group, respectively. **C,D** show high and low risk examples for the non-CMP-IV group, respectively.

**Figure 6:**
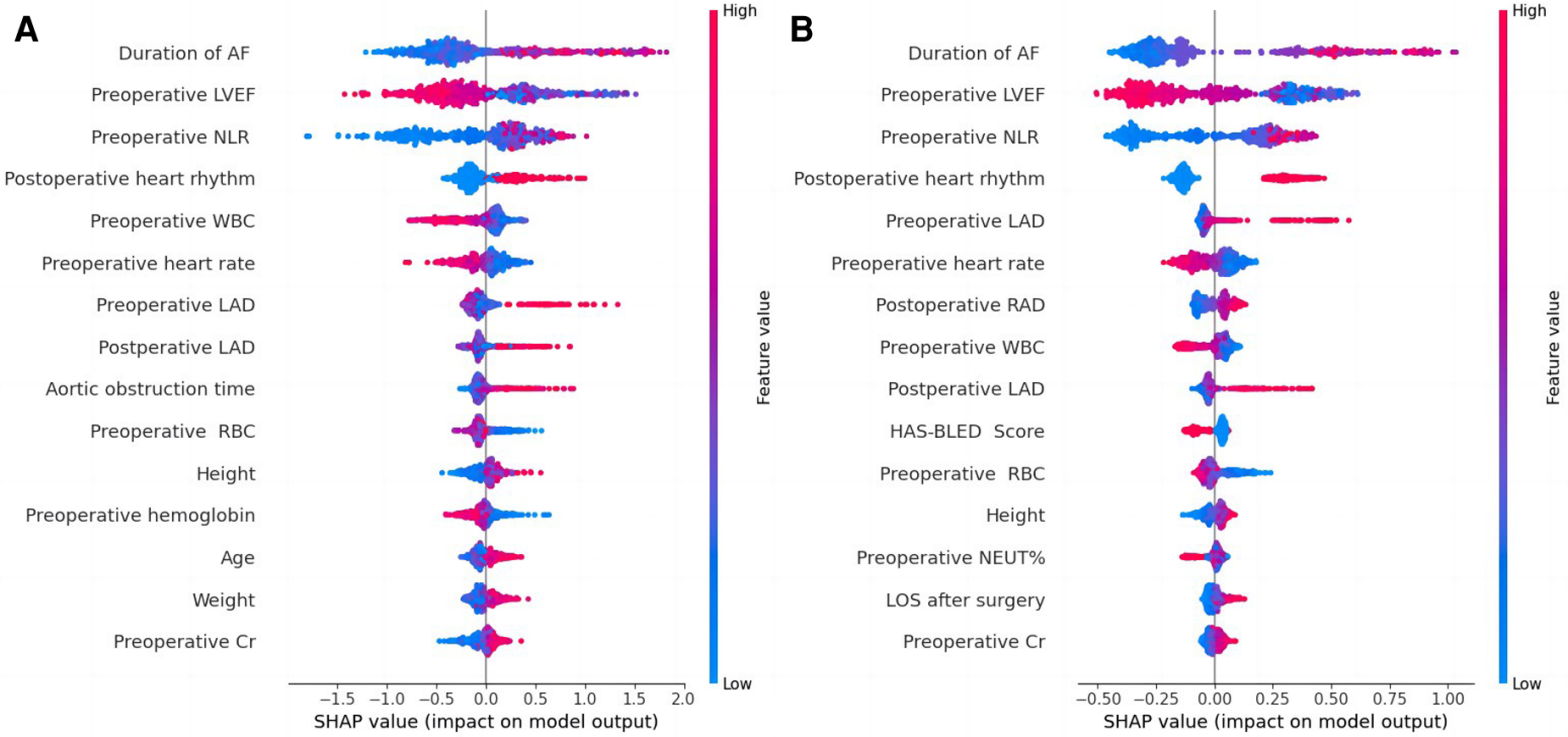
SHAP summary plots based on XGBoost model output (**A**) and catBoost model output (**B**) from the CMP-IV group. SHAP summary plots are used to visualize the impact of each feature on the model output. The plot includes a list of features on the left side and the corresponding feature values on the right side. The middle part of the plot is a cluster of coloured dots, where the red dots represent features that have a positive impact on the model output, the blue dots represent features that have a negative impact, and the purple dots represent features that have a neutral impact. The size of the dots represents the magnitude of the impact of each feature, with larger dots indicating a greater impact. The influence of each feature on the outcome can be discerned by comparing the position of the dots to the reference line.

Four machine learning models (LightGBM, XGBoost, RF and CatBoost) were selected to analyze the risk factors for AF recurrence in the CMP-IV group and Non-CMP-IV group based on the model evaluation. The top 20 feature importance of the 4 models in the CMP-IV group is shown in Figure 3. The feature importance of the 4 models for the Non-CMP-IV group is shown in Figure 4. The SHAP force plots in Figure 5 allow us to evaluate the risk of AF recurrence for individual samples, which shows high-risk and low-risk examples of AF recurrence in both the CMP-IV and Non-CMP-IV groups. Figure 6 shows the SHAP summary diagrams for the analysis of the XGBoost model and the Catboost model.

## 4. Discussion

We conducted a retrospective cohort study of the CMP-IV group and the Non-CMP-IV group and found that the CMP-IV resulted in a significantly higher rate of sinus rhythm maintenance at 5 years (82.13% in the CMP-IV group compared to 13.40% in the Non-CMP-IV group). This suggests that CMP-IV treatment for AF is highly effective and consistent with previous literature reports (34, 35). Left atrial appendage ligation can reduce the risk of thrombosis formation (36–38). Kawamura et al. found that left atrial appendage ligation may lead to a decrease in atrial dispersion, thereby improving the rate of sinus rhythm maintenance (39). We found that most patients in the Non-CMP-IV group who underwent valve surgery combined with left atrial appendage ligation experienced the recurrence of AF in the early postoperative period. A small number of patients in the Non-CMP-IV group regained sinus rhythm, which may be related to drug treatment and paroxysmal AF (40–42).

We successfully developed 10 machine learning models in each group. CatBoost, XGBoost, LightGBM, and Random Forest outperformed the others in predicting and classifying this data set. Using feature importance analysis and SHAP interpretability analysis, we identified several risk factors for AF recurrence by ranking the obtained features according to their importance and the number of occurrences in each model. In the CMP-IV group, the most significant risk factors were the duration of AF and preoperative LVEF. Other important risk factors included postoperative heart rhythm, preoperative LAD, preoperative NLR, preoperative heart rate, and preoperative WBC. In the Non-CMP-IV group, the most important risk factors were preoperative LAD and postoperative heart rate. Other potentially influential risk factors included preoperative WBC and preoperative NEUT%, preoperative ALT, and postoperative LVEF.

In our study, all four of our machine learning models in the CMP-IV group found that the duration of AF is the most important feature for AF recurrence. This is similar to a Meta-analysis result of Chew et al. which found that the duration between the first diagnosis of AF and ablation, or diagnosis-to-ablation time (DAT), is associated with AF recurrence following catheter ablation (43). Andrade et al. also found that AF episode duration is associated with post-AF outcomes, in which longer episodes are linked to higher AF burden (44). Our study further suggests that the risk of AF recurrence increases significantly with a prolonged duration of AF. We found that preoperative LVEF is also an important risk factor for AF recurrence in the CMP-IV group. The SHAP summary plot of the two models showed that as preoperative LVEF decreases, the risk of AF recurrence increases. While this feature was not identified in previous studies, its importance in our models ranked second among all four models, indicating a high level of influence on the outcome. One possible reason is that the use of machine learning methods allowed us to identify novel risk factors that were not previously known to be associated with AF recurrence. This provided us with a deeper understanding of the relationships between the various risk factors and AF recurrence. Further research is needed to confirm the role of preoperative LVEF in AF recurrence. Our multiple models also found that LAD is closely related to AF recurrence. The SHAP interpreter clearly showed that the risk of AF recurrence increases with increasing preoperative or intraoperative LAD, which is consistent with previous studies (45–47). The feature importance analysis of the XGBoost and Catboost models found that combined tricuspid valve surgery is also a risk factor for AF recurrence. Previous studies have found that long-term AF is associated with functional tricuspid regurgitation, and severe tricuspid regurgitation may be associated with AF (48, 49), which suggests that preoperative tricuspid insufficiency may be associated with the risk of AF recurrence.

WBC has been found to correlate with cardiovascular risk (50), we also found that preoperative neutrophil-lymphocyte ratio (NLR) was significantly associated with AF recurrence after CMP-IV. NLR is linked to cardiovascular risk and can predict the prognosis of various clinical diseases (51, 52), including hypertension and heart failure (53, 54). High NLR could potentially be used as a predictor for AF recurrence (55, 56), which is in line with our machine learning model's further findings, as the SHAP summary plot shows that as NLR increases, the risk of AF recurrence also increases. In addition, our study found that early postoperative AF recurrence is correlated with final AF recurrence. Specifically, the presence of postoperative early recurrence, which may be related to the postoperative excessive inflammatory response (57, 58), significantly increases the risk of late AF recurrence. This finding is consistent with previous research on radiofrequency catheter ablation by Kim et al. (59). Prothrombin time (PT) and international normalized ratio (INR) are used to measure the effectiveness of anticoagulants in patients with AF. Combined with the analysis results of multiple models, Has-bled (60), EuroSCore II (61) and CHA_2_DS_2_VASc (62) which are commonly used surgical scoring tools, were not found to be significant risk factors in predicting AF recurrence in this study. Additionally, gender, age, and the main type of valve surgery were not found to be significant risk factors.

In the feature importance analysis of the Non-CMP-IV group, we found that the LAD was consistently identified as the most important factor associated with AF recurrence in all four models. However, as most patients in this group still had AF postoperatively and AF was detected early, the characteristic value gaps for most features were small, leading to potentially weakened correlations between these risk factors and AF recurrence.

Our study found that the four groups of models had high consistency in identifying the most influential risk factors for AF recurrence, but the results for risk factors with lower feature importance varied among the models. Besides, the differences in feature importance rankings can be attributed to the different algorithms and approaches used to calculate feature importance. This suggests that the machine learning models were effective in identifying key risk factors, but there may be room for further optimization in the assessment of overall risk factors. Similar to the SHAP force plot analysis based on a single sample in Figure 5, it is helpful for individualized risk assessment of AF recurrence for each patient. Previous studies on risk factors for recurrence of atrial fibrillation after CMP-IV have been conducted (14, 15, 63), but they were based on traditional statistical analyses. Our study expanded the sample size and length of follow-up compared to previous studies and used machine learning algorithms to visualize the risk factors for recurrence of atrial fibrillation for the overall study and for individual patients.

Our study has a few limitations. One is that the sample size of our study may not be sufficient to fully capture the complexity of AF recurrence. Additionally, our study was conducted at two hospitals and may not be generalizable to other settings. Furthermore, the effectiveness of machine learning techniques depends on the quality and relevance of input features. In this study, we only considered a limited number of features and did not analyze more detailed intraoperative data or evaluate the transmurality of ablation in patients. There may be other risk factors for AF recurrence that have not been identified. The clinical application of this study will help to establish a predictive model for AF recurrence after CMP-IV. This will serve as precision therapy by helping to individualize patient treatment. It will allow surgeons to improve treatment plans in a timely manner, thereby improving the prognosis of patients. Further research can continue to study individualized risk factors of AF recurrence with larger-scale data sets and specific values, such as developing an individualized assessment system for AF recurrence to help surgeons to assess the prognosis of AF surgical treatment based on individual patient data.

## 5. Conclusion

In conclusion, our study found that Cox-Maze IV procedure is effective in treating AF and maintaining sinus rhythm in patients with AF and valvular disease. We have successfully developed multiple machine learning models and identified several clinical risk factors for AF recurrence, including duration of AF, preoperative LVEF, postoperative heart rhythm, preoperative LAD, preoperative NLR, preoperative heart rate, postoperative LAD, preoperative WBC and postoperative AST. CatBoost, XGBoost, LightGBM, and Random Forest performed the best in predicting AF recurrence in this data set. Machine learning models can be used to identify risk factors and may be useful in predicting and preventing AF recurrence in clinical practice. Further studies are needed to confirm our findings and to determine the generalizability of these results.

## Data Availability

The raw data supporting the conclusions of this article will be made available by the authors, without undue reservation.

## References

[1] Gillinov AM Bagiella E Moskowitz AJ Raiten JM Groh MA Bowdish ME Rate control versus rhythm control for atrial fibrillation after cardiac surgery. N Engl J Med. (2016) 374(20):1911–21. 10.1056/NEJMoa1602002 27043047 PMC4908812

[2] Miyasaka Y Barnes ME Gersh BJ Cha SS Bailey KR Abhayaratna WP Secular trends in incidence of atrial fibrillation in olmsted county, Minnesota, 1980 to 2000, and implications on the projections for future prevalence. Circulation. (2006) 114(2):119–25. 10.1161/CIRCULATIONAHA.105.595140 16818816

[3] Paciaroni M Agnelli G Micheli S Caso V. Efficacy and safety of anticoagulant treatment in acute cardioembolic stroke: a meta-analysis of randomized controlled trials. Stroke. (2007) 38(2):423–30. 10.1161/01.STR.0000254600.92975.1f 17204681

[4] von Kummer R Broderick JP Campbell BC Demchuk A Goyal M Hill MD The Heidelberg bleeding classification: classification of bleeding events after ischemic stroke and reperfusion therapy. Stroke. (2015) 46(10):2981–6. 10.1161/STROKEAHA.115.010049 26330447

[5] Ruddox V Sandven I Munkhaugen J Skattebu J Edvardsen T Otterstad JE. Atrial fibrillation and the risk for myocardial infarction, all-cause mortality and heart failure: a systematic review and meta-analysis. Eur J Prev Cardiol. (2017) 24(14):1555–66. 10.1177/2047487317715769 28617620 PMC5598874

[6] Bohm M Ezekowitz MD Connolly SJ Eikelboom JW Hohnloser SH Reilly PA Changes in renal function in patients with atrial fibrillation: an analysis from the RE-LY trial. J Am Coll Cardiol. (2015) 65(23):2481–93. 10.1016/j.jacc.2015.03.577 26065986

[7] Garcia-Villarreal OA. The cox-maze procedure: rules not to be broken. Ann Thorac Surg. (2021) 112(6):2109. 10.1016/j.athoracsur.2021.02.026 33662310

[8] Ruaengsri C Schill MR Khiabani AJ Schuessler RB Melby SJ Damiano RJ Jr. The cox-maze IV procedure in its second decade: still the gold standard? Eur J Cardiothorac Surg. (2018) 53(suppl_1):i19–25. 10.1093/ejcts/ezx326 29590383 PMC6018688

[9] Ad N Henry L Hunt S Holmes SD. The outcome of the cox maze procedure in patients with previous percutaneous catheter ablation to treat atrial fibrillation. Ann Thorac Surg. (2011) 91(5):1371–7; discussion 1377. 10.1016/j.athoracsur.2011.01.026 21457939

[10] Robertson JO Saint LL Leidenfrost JE Damiano RJ Jr. Illustrated techniques for performing the cox-maze IV procedure through a right mini-thoracotomy. Ann Cardiothorac Surg. (2014) 3(1):105–16. 10.3978/j.issn.2225-319X.2013.12.11 24516807 PMC3904342

[11] Calkins H Kuck KH Cappato R Brugada J Camm AJ Chen SA 2012 HRS/EHRA/ECAS expert consensus statement on catheter and surgical ablation of atrial fibrillation: recommendations for patient selection, procedural techniques, patient management and follow-up, definitions, endpoints, and research trial design. Europace. (2012) 14(4):528–606. 10.1093/europace/eus027 22389422

[12] Winkle RA Mead RH Engel G Patrawala RA. Long-term results of atrial fibrillation ablation: the importance of all initial ablation failures undergoing a repeat ablation. Am Heart J. (2011) 162(1):193–200. 10.1016/j.ahj.2011.04.013 21742108

[13] Kim JS Lee SA Park JB Chee HK Chung JW. Preoperative risk factor analysis of postoperative stroke after cox-maze procedure with mitral valve repair. BMC Cardiovasc Disord. (2014) 14:116. 10.1186/1471-2261-14-116 25212180 PMC4169863

[14] Takagaki M Yamaguchi H Ikeda N Yamakage H Nakamura H Kadowaki T Risk factors for atrial fibrillation recurrence after cox maze IV performed without pre-exclusion. Ann Thorac Surg. (2020) 109(3):771–9. 10.1016/j.athoracsur.2019.07.016 31472135

[15] Damiano RJ Jr. Schwartz FH Bailey MS Maniar HS Munfakh NA Moon MR The cox maze IV procedure: predictors of late recurrence. J Thorac Cardiovasc Surg. (2011) 141(1):113–21. 10.1016/j.jtcvs.2010.08.067 21168019 PMC3035158

[16] Ij H. Statistics versus machine learning. Nat Methods. (2018) 15(4):233. 10.1038/nmeth.4642 30100822 PMC6082636

[17] Rajula HSR Verlato G Manchia M Antonucci N Fanos V. Comparison of conventional statistical methods with machine learning in medicine: diagnosis, drug development, and treatment. Medicina. (2020) 56(9):455. 10.3390/medicina56090455 32911665 PMC7560135

[18] Jordan MI Mitchell TM. Machine learning: trends, perspectives, and prospects. Science. (2015) 349(6245):255–60. 10.1126/science.aaa8415 26185243

[19] Verma AA Murray J Greiner R Cohen JP Shojania KG Ghassemi M Implementing machine learning in medicine. Can Med Assoc J. (2021) 193(34):E1351–7. 10.1503/cmaj.202434 35213323 PMC8432320

[20] Peiffer-Smadja N Rawson TM Ahmad R Buchard A Georgiou P Lescure FX Machine learning for clinical decision support in infectious diseases: a narrative review of current applications. Clin Microbiol Infect. (2020) 26(5):584–95. 10.1016/j.cmi.2019.09.009 31539636

[21] van Kooten RT Bahadoer RR Ter Buurkes de Vries B Wouters M Tollenaar R Hartgrink HH Conventional regression analysis and machine learning in prediction of anastomotic leakage and pulmonary complications after esophagogastric cancer surgery. J Surg Oncol. (2022) 126(3):490–501. 10.1002/jso.26910 35503455 PMC9544929

[22] Wagner M Brandenburg JM Bodenstedt S Schulze A Jenke AC Stern A Surgomics: personalized prediction of morbidity, mortality and long-term outcome in surgery using machine learning on multimodal data. Surg Endosc. (2022) 36(11):8568–91. 10.1007/s00464-022-09611-1 36171451 PMC9613751

[23] Noble WS. What is a support vector machine? Nat Biotechnol. (2006) 24(12):1565–7. 10.1038/nbt1206-1565 17160063

[24] Wright RE. Logistic regression (1995).

[25] Chen T Guestrin C. Xgboost: a scalable tree boosting system. Proceedings of the 22nd acm sigkdd international conference on knowledge discovery and data mining (2016).

[26] Breiman L. Random forests. Mach Learn. (2001) 45(1):5–32. 10.1023/A:1010933404324

[27] Prokhorenkova L Gusev G Vorobev A Dorogush AV Gulin A. CatBoost: unbiased boosting with categorical features. Advances in neural information processing systems (2018). p. 31.

[28] Schapire RE. Explaining adaboost. In: Schölkopf B, editor. Empirical inference. Berlin: Springer (2013). p. 37–52.

[29] Lee T-H Ullah A Wang R. Bootstrap aggregating and random forest. In: Fuleky P, Kilian L, editors. Macroeconomic forecasting in the era of big data. Cham: Springer (2020). p. 389–429.

[30] Friedman JH. Stochastic gradient boosting. Comput Stat Data Anal. (2002) 38(4):367–78. 10.1016/S0167-9473(01)00065-2

[31] Ke G Meng Q Finley T Wang T Chen W Ma W Lightgbm: a highly efficient gradient boosting decision tree. Adv Neural Inf Process Syst. (2017) 30:3149–57. 10.5555/3294996.3295074

[32] Tang J Deng C Huang GB. Extreme learning machine for multilayer perceptron. IEEE Trans Neural Netw Learn Syst. (2016) 27(4):809–21. 10.1109/TNNLS.2015.2424995 25966483

[33] DeLong ER DeLong DM Clarke-Pearson DL. Comparing the areas under two or more correlated receiver operating characteristic curves: a nonparametric approach. Biometrics. (1988) 44(3):837–45. 10.2307/2531595 3203132

[34] Khiabani AJ MacGregor RM Bakir NH Manghelli JL Sinn LA Maniar HS The long-term outcomes and durability of the cox-maze IV procedure for atrial fibrillation. J Thorac Cardiovasc Surg. (2022) 163(2):629–641.e7. 10.1016/j.jtcvs.2020.04.100 32563577 PMC9810144

[35] Philpott JM Zemlin CW Cox JL Stirling M Mack M Hooker RL The ABLATE trial: safety and efficacy of cox maze-IV using a bipolar radiofrequency ablation system. Ann Thorac Surg. (2015) 100(5):1541–6; discussion 1547–8. 10.1016/j.athoracsur.2015.07.006 26387721

[36] Kim R Baumgartner N Clements J. Routine left atrial appendage ligation during cardiac surgery may prevent postoperative atrial fibrillation-related cerebrovascular accident. J Thorac Cardiovasc Surg. (2013) 145(2):582–9; discussion 589. 10.1016/j.jtcvs.2012.10.016 23137520

[37] Ando M Funamoto M Cameron DE Sundt TM. Concomitant surgical closure of left atrial appendage: a systematic review and meta-analysis. J Thorac Cardiovasc Surg. (2018) 156(3):1071–1080.e2. 10.1016/j.jtcvs.2018.03.017 29628346

[38] Melduni RM Schaff HV Lee HC Gersh BJ Noseworthy PA Bailey KR Impact of left atrial appendage closure during cardiac surgery on the occurrence of early postoperative atrial fibrillation, stroke, and mortality: a propensity score-matched analysis of 10 633 patients. Circulation. (2017) 135(4):366–78. 10.1161/CIRCULATIONAHA.116.021952 27903589 PMC5469206

[39] Kawamura M Scheinman MM Lee RJ Badhwar N. Left atrial appendage ligation in patients with atrial fibrillation leads to a decrease in atrial dispersion. J Am Heart Assoc. (2015) 4(5):e001581. 10.1161/JAHA.114.001581 25977469 PMC4599401

[40] Sardar MR Saeed W Kowey PR. Antiarrhythmic drug therapy for atrial fibrillation. Heart Fail Clin. (2016) 12(2):205–21. 10.1016/j.hfc.2015.08.017 26968666

[41] Poole JE Bahnson TD Monahan KH Johnson G Rostami H Silverstein AP Recurrence of atrial fibrillation after catheter ablation or antiarrhythmic drug therapy in the CABANA trial. J Am Coll Cardiol. (2020) 75(25):3105–18. 10.1016/j.jacc.2020.04.065 32586583 PMC8064404

[42] Zimetbaum P. Antiarrhythmic drug therapy for atrial fibrillation. Circulation. (2012) 125(2):381–9. 10.1161/CIRCULATIONAHA.111.019927 22249528

[43] Chew DS Black-Maier E Loring Z Noseworthy PA Packer DL Exner DV Diagnosis-to-ablation time and recurrence of atrial fibrillation following catheter ablation: a systematic review and meta-analysis of observational studies. Circ Arrhythm Electrophysiol. (2020) 13(4):e008128. 10.1161/CIRCEP.119.008128 32191539 PMC7359927

[44] Andrade JG Deyell MW Verma A Macle L Champagne J Leong-Sit P Association of atrial fibrillation episode duration with arrhythmia recurrence following ablation: a secondary analysis of a randomized clinical trial. JAMA Netw Open. (2020) 3(7):e208748. 10.1001/jamanetworkopen.2020.8748 32614422 PMC7333024

[45] Minami K Kabata D Kakuta T Fukushima S Fujita T Shintani A U-shaped association between intraoperative net fluid balance and risk of postoperative recurrent atrial tachyarrhythmia among patients undergoing the cryo-maze procedure: an observational study. J Cardiothorac Vasc Anesth. (2021) 35(8):2392–6. 10.1053/j.jvca.2020.10.023 33158709

[46] Hwang SK Yoo JS Kim JB Jung SH Choo SJ Chung CH Long-term outcomes of the maze procedure combined with mitral valve repair: risk of thromboembolism without anticoagulation therapy. Ann Thorac Surg. (2015) 100(3):840–3; discussion 843–4. 10.1016/j.athoracsur.2015.02.073 26116476

[47] Jin X Pan J Wu H Xu D. Are left ventricular ejection fraction and left atrial diameter related to atrial fibrillation recurrence after catheter ablation?: a meta-analysis. Medicine. (2018) 97(20):e10822. 10.1097/MD.0000000000010822 29768386 PMC5976293

[48] Muraru D Caravita S Guta AC Mihalcea D Branzi G Parati G Functional tricuspid regurgitation and atrial fibrillation: which comes first, the chicken or the egg? CASE. (2020) 4(5):458–63. 10.1016/j.case.2020.04.011 33117949 PMC7581628

[49] Bak M Jeong DS Park S-J Park B Seo JH Park I Predictor of atrial fibrillation recurrence in patients who underwent a tricuspid valve operation with modified cox maze procedure. Echocardiography. (2022) 39(3):447–56. 10.1111/echo.15315 35165935

[50] Horne BD Anderson JL John JM Weaver A Bair TL Jensen KR Which white blood cell subtypes predict increased cardiovascular risk? J Am Coll Cardiol. (2005) 45(10):1638–43. 10.1016/j.jacc.2005.02.054 15893180

[51] Khiabani AJ Schuessler RB Damiano RJ Jr. Surgical ablation of atrial fibrillation in patients with heart failure. J Thorac Cardiovasc Surg. (2021) 162(4):1100–5. 10.1016/j.jtcvs.2020.05.125 32948298 PMC9680982

[52] Yayla C Akboga MK Gayretli Yayla K Ertem AG Efe TH Sen F A novel marker of inflammation in patients with slow coronary flow: lymphocyte-to-monocyte ratio. Biomark Med. (2016) 10(5):485–93. 10.2217/bmm-2016-0022 27089433

[53] Fici F Celik T Balta S Iyisoy A Unlu M Demitkol S Comparative effects of nebivolol and metoprolol on red cell distribution width and neutrophil/lymphocyte ratio in patients with newly diagnosed essential hypertension. J Cardiovasc Pharmacol. (2013) 62(4):388–93. 10.1097/FJC.0b013e31829f716a 23921307

[54] Uthamalingam S Patvardhan EA Subramanian S Ahmed W Martin W Daley M Utility of the neutrophil to lymphocyte ratio in predicting long-term outcomes in acute decompensated heart failure. Am J Cardiol. (2011) 107(3):433–8. 10.1016/j.amjcard.2010.09.039 21257011

[55] Silberman S Fink D. Neutrophil to lymphocyte ratio and risk of atrial fibrillation. Am J Cardiol. (2019) 123(11):1889. 10.1016/j.amjcard.2019.03.033 31000138

[56] Min K Kwon S Cho S-Y Choi WJ Park S-U Jung W-S Atrial fibrillation is strongly associated with the neutrophil to lymphocyte ratio in acute ischemic stroke patients: a retrospective study. J Clin Lab Anal. (2017) 31(2):e22041. 10.1002/jcla.22041 27558309 PMC6817156

[57] Wei Y Bao Y Lin C Xie Y Luo Q Zhang N Early recurrence after cryoballoon versus radiofrequency ablation for paroxysmal atrial fibrillation: mechanism and implication in long-term outcome. BMC Cardiovasc Disord. (2022) 22(1):400. 10.1186/s12872-022-02816-1 36071377 PMC9450458

[58] McCabe JM Smith LM Tseng ZH Badhwar N Lee BK Lee RJ Protracted CRP elevation after atrial fibrillation ablation. Pacing Clin Electrophysiol. (2008) 31(9):1146–51. 10.1111/j.1540-8159.2008.01155.x 18834466 PMC2596767

[59] Kim YG Boo KY Choi J-I Choi YY Choi HY Roh S-Y Early recurrence is reliable predictor of late recurrence after radiofrequency catheter ablation of atrial fibrillation. JACC Clin Electrophysiol. (2021) 7(3):343–51. 10.1016/j.jacep.2020.09.029 33516711

[60] Pisters R Lane DA Nieuwlaat R de Vos CB Crijns HJ Lip GY. A novel user-friendly score (HAS-BLED) to assess 1-year risk of major bleeding in patients with atrial fibrillation: the euro heart survey. Chest. (2010) 138(5):1093–100. 10.1378/chest.10-0134 20299623

[61] Nashef SAM Roques F Sharples LD Nilsson J Smith C Goldstone AR EuroSCORE II. Eur J Cardiothorac Surg. (2012) 41(4):734–45. 10.1093/ejcts/ezs043. 22378855

[62] Camm AJ Kirchhof P Lip GYH Schotten U Savelieva I Ernst S Guidelines for the management of atrial fibrillation: the task force for the management of atrial fibrillation of the European society of cardiology (ESC). Eur Heart J. (2010) 31(19):2369–429. 10.1093/eurheartj/ehq278 20802247

[63] Kakuta T Fukushima S Minami K Saito T Kawamoto N Tadokoro N Novel risk score for predicting recurrence of atrial fibrillation after the cryo-maze procedure. Eur J Cardiothorac Surg. (2021) 59(6):1218–25. 10.1093/ejcts/ezaa468 33550393

