# Supplementary material for: Machine learning-based analysis of risk factors for atrial fibrillation recurrence after Cox-Maze IV procedure in patients with atrial fibrillation and chronic valvular disease: A retrospective cohort study with a control group
Source: Front Cardiovasc Med. 2023 Mar 24;10:1140670. doi: 10.3389/fcvm.2023.1140670 (PMC10079913; doi:10.3389/fcvm.2023.1140670)
Supplement: Supplementary file 1 [file Datasheet1.docx]

Supplementary Material

Machine learning-based analysis of risk factors for atrial fibrillation recurrence after Cox-Maze IV procedure in patients with atrial fibrillation and chronic valvular disease: A retrospective cohort study with a control group

Zenan Jiang, Long Song, Chunshui Liang, Hao Zhang, Haoyu Tan, Yaqin Sun, Ruikang Guo, Liming Liu*

*** Correspondence:** Liming Liu: liulimingjia@csu.edu.cn

# Inclusion criteria

From January 1, 2012, to December 31, 2019, patients with chronic valvular disease and AF at the Second Xiangya Hospital of Central South University and Xinqiao Hospital Affiliated to Army Medical University:

1) The age at the time of surgery is over 35 years old, male or female;

2) Valve history of more than 3 years at the time of surgery;

3) The history of atrial fibrillation at the time of surgery meets any of the following:

a. Persistent atrial fibrillation (atrial fibrillation lasting more than 7 days or requiring the use of drugs or electrical cardioversion to convert the heart rhythm) for more than 3 years;

b. Long-term persistent atrial fibrillation (atrial fibrillation has lasted for more than 1 year when it was decided to take cardiac rhythm control measures);

c. Paroxysmal atrial fibrillation with ECG records;

4) Left ventricular end-diastolic diameter (left ventricular long-axis view) ≤ 75mm; left atrium anteroposterior diameter (left ventricular long-axis view) ≤ 75mm;

5) EF value ≥ 40% at the time of surgery;

6) The clinical diagnosis meets any of the following items:

a. Rheumatic mitral stenosis and regurgitation, with or without tricuspid regurgitation;

b. Rheumatic aortic stenosis and regurgitation, with or without tricuspid regurgitation;

c. Rheumatic mitral valve, aortic valve stenosis and regurgitation, with or without tricuspid regurgitation;

d. Non-rheumatic mitral and (or) aortic regurgitation, with or without tricuspid regurgitation;

7) The patient signs the informed consent.

# Exclusion criteria

1) Patients who need coronary artery bypass grafting at the same time;

2) Emergency surgery patients;

3) Massive thrombus in the left atrium;

4) Life expectancy ≤ 3 years;

5) Cancer patients who have been diagnosed and treated;

6) Any factors that may limit the compliance of the intervention as determined by the research team;

7) Failure to obtain the informed consent of the subjects;

8) Participating in other interventional research;

9) Pregnant women or pregnancy plans during the study period.

# Supplementary Table 1: Baseline, hospitalization and follow-up characteristics of the patients in the CMP-IV group.

| n |  | Total (n=555) | AF non-recurrence (n=438) | AF recurrence (n=117) | P |
| --- | --- | --- | --- | --- | --- |
| Gender, n(%) | Female | 370 (66.667) | 297 (67.654) | 73 (62.93) | 0.337 |
|  | male | 185 (33.333) | 142 (32.346) | 43 (37.069) |  |
| Age, years |  | 57.948 ± 7.964 | 57.722 ± 8.024 | 58.802 ± 7.670 | 0.195 |
| Duration of AF, years |  | 3.800 [2.800,5.000] | 3.300 [2.000,4.300] | 4.800 [3.700,6.100] | <0.001 |
| Height, cm |  | 158.871 ± 8.056 | 158.448 ± 7.980 | 160.472 ± 8.141 | 0.016 |
| Weight, cm |  | 58.744 ± 10.302 | 58.547 ± 10.339 | 59.491 ± 10.127 | 0.381 |
| SBP, mmHg |  | 113.881 ± 16.162 | 113.699 ± 16.068 | 114.569 ± 16.493 | 0.607 |
| DBP, mmHg |  | 71.694 ± 11.037 | 71.613 ± 11.046 | 72.000 ± 10.997 | 0.737 |
| BMI, kg/m^2^ |  | 23.193 ± 3.126 | 23.245 ± 3.214 | 22.994 ± 2.756 | 0.443 |
| Preoperative heart rate, bpm |  | 91.063 ± 20.302 | 91.934 ± 20.699 | 87.767 ± 18.351 | 0.049 |
| Preoperative LAD, mm |  | 52.048 ± 8.498 | 51.216 ± 7.707 | 55.194 ± 10.402 | <0.001 |
| Preoperative RAD, mm |  | 38.944 ± 6.562 | 38.543 ± 6.128 | 40.462 ± 7.809 | 0.016 |
| Preoperative LVD, mm |  | 49.930 ± 8.118 | 49.930 ± 8.188 | 49.933 ± 7.847 | 0.997 |
| Preoperative RVD, mm |  | 36.896 ± 6.024 | 36.720 ± 5.851 | 37.563 ± 6.597 | 0.181 |
| Preoperative LVEF, % |  | 61.441 ± 8.108 | 62.079 ± 8.325 | 59.026 ± 6.698 | <0.001 |
| Preoperative hypertension, n (%) |  | 50 (9.009) | 40 (9.112) | 10 (8.621) | 0.870 |
| Preoperative diabetes, n (%) |  | 17 (3.063) | 11 (2.506) | 6 (5.172) | 0.138 |
| Preoperative CHD, n (%) |  | 42 (7.568) | 30 (6.834) | 12 (10.345) | 0.203 |
| History of preoperative cerebral infarction, n (%) |  | 34 (6.126) | 27 (6.150) | 7 (6.034) | 0.866 |
| Preoperative pulmonary hypertension, n (%) |  | 149 (26.847) | 121 (27.563) | 28 (24.138) | 0.459 |
| Smoking or drinking, n (%) |  | 24 (4.324) | 18 (4.100) | 6 (5.172) | 0.614 |
| HAS-BLED Score, n (%) | 0 | 390 (70.270) | 300 (68.337) | 90 (77.586) | 0.149 |
|  | 1 | 146 (26.306) | 123 (28.018) | 23 (19.828) |  |
|  | 2 | 17 (3.063) | 15 (3.417) | 2 (1.724) |  |
|  | 4 | 2 (0.360) | 1 (0.228) | 1 (0.862) |  |
| CHA2DS2-VASc Score, n (%) | 0 | 142 (25.586) | 109 (24.829) | 33 (28.448) | 0.395 |
|  | 1 | 334 (60.180) | 263 (59.909) | 71 (61.207) |  |
|  | 2 | 40 (7.207) | 35 (7.973) | 5 (4.310) |  |
|  | 3 | 35 (6.306) | 30 (6.834) | 5 (4.310) |  |
|  | 4 | 2 (0.360) | 1 (0.228) | 1 (0.862) |  |
|  | 5 | 2 (0.360) | 1 (0.228) | 1 (0.862) |  |
| Euro Score Ⅱ score |  | 1.630 ± 0.936 | 1.635 ± 0.975 | 1.609 ± 0.766 | 0.787 |
| Preoperative WBC, 10^12^/L |  | 6.281 ± 1.944 | 6.369 ± 2.023 | 5.950 ± 1.565 | 0.017 |
| Preoperative NEUT%, % |  | 60.280 ± 10.153 | 60.339 ± 10.188 | 60.059 ± 10.016 | 0.792 |
| Preoperative RBC, 10^12^/L |  | 4.754 ± 1.332 | 4.777 ± 1.315 | 4.668 ± 1.391 | 0.434 |
| Preoperative hemoglobin, g/L |  | 129.652 ± 15.821 | 129.952 ± 15.622 | 128.517 ± 16.504 | 0.386 |
| Preoperative neutrophil, 10^9^/L |  | 4.439 ± 1.710 | 4.382 ± 1.732 | 4.655 ± 1.608 | 0.124 |
| Preoperative lymphocyte, 10^9^/L |  | 1.874 ± 0.818 | 1.904 ± 0.833 | 1.761 ± 0.745 | 0.093 |
| Preoperative NLR |  | 2.342 [1.647,3.419] | 2.299 [1.581,3.247] | 2.550 [1.838,3.895] | 0.005 |
| Preoperative PLT, 10^^9^/l |  | 191.587 ± 74.388 | 190.631 ± 72.897 | 195.207 ± 79.677 | 0.557 |
| Preoperative INR |  | 1.134 ± 0.363 | 1.132 ± 0.381 | 1.138 ± 0.283 | 0.878 |
| Preoperative PT, s |  | 12.985 ± 4.282 | 12.984 ± 4.538 | 12.991 ± 3.130 | 0.988 |
| Preoperative AST, U/L |  | 29.900 [20.900,50.700] | 28.400 [20.660,49.990] | 32.630 [21.600,53.520] | 0.138 |
| Preoperative ALT, U/L |  | 26.400 [17.300,42.200] | 26.400 [17.200,40.800] | 26.400 [18.700,45.200] | 0.29 |
| Preoperative Creatinine, µmol/l |  | 72.800 [60.200,90.800] | 72.100 [59.500,90.300] | 77.600 [63.800,92.000] | 0.187 |
| Preoperative BUN, mmol/l |  | 6.241 ± 2.618 | 6.227 ± 2.579 | 6.293 ± 2.758 | 0.810 |
| Preoperative Tbil, μmol/L |  | 14.700 [10.600,21.000] | 14.900 [10.600,21.800] | 14.200 [10.200,19.100] | 0.387 |
| Preoperative NYHA, n (%) | I＆II | 32 (5.766) | 25 (5.695) | 7 (6.034) | 0.797 |
|  | III | 447 (80.541) | 356 (81.093) | 91 (78.448) |  |
|  | IV | 76 (13.694) | 58 (13.212) | 18 (15.517) |  |
| Type of Surgery, n (%) | AVR | 6 (1.081) | 6 (1.367) | 0 (0.000) | 0.293 |
|  | DVR | 229 (41.261) | 185 (42.141) | 44 (37.931) |  |
|  | MVR | 299 (53.874) | 231 (52.620) | 68 (58.621) |  |
|  | TVP only | 3 (0.541) | 1 (0.228) | 2 (1.724) |  |
|  | MVP only | 17 (3.063) | 15 (3.417) | 2 (1.724) |  |
|  | TVR only | 1 (0.180) | 1 (0.228) | 0 (0.000) |  |
| MVP combined, n (%) |  | 20 (3.604) | 18 (4.100) | 2 (1.724) | 0.222 |
| TVP combined, n (%) |  | 479 (86.306) | 379 (86.333) | 100 (86.207) | 0.972 |
| TVR combined, n (%) |  | 4 (0.721) | 4 (0.911) | 0 (0.000) | 0.304 |
| CBP time, min |  | 99.000 [80.000,120.000] | 98.000 [80.000,120.000] | 101.000 [81.000,120.000] | 0.475 |
| Aortic obstruction time, min |  | 63.000 [48.000,78.000] | 64.000 [48.000,77.000] | 63.000 [48.000,80.000] | 0.605 |
| Left atrial thrombus, n (%) |  | 52 (9.369) | 39 (8.884) | 13 (11.207) | 0.445 |
| Postoperative heart rhythm, n (%) | Non-sinus rhythm | 168 (30.270) | 107 (24.374) | 61 (52.586) | <0.001 |
| Postoperative heart rate, bpm |  | 84.784 ± 14.933 | 84.893 ± 14.733 | 84.371 ± 15.661 | 0.738 |
| Postoperative LAD, mm |  | 42.808 ± 6.889 | 42.140 ± 6.353 | 45.339 ± 8.139 | <0.001 |
| Postoperative RAD, mm |  | 35.841 ± 4.743 | 35.456 ± 4.312 | 37.298 ± 5.881 | <0.001 |
| Postoperative LVD, mm |  | 45.654 ± 5.670 | 45.468 ± 5.598 | 46.357 ± 5.880 | 0.134 |
| Postoperative RVD, mm |  | 34.765 ± 4.027 | 34.502 ± 3.637 | 35.760 ± 5.124 | 0.003 |
| Postoperative LVEF, % |  | 64.716 ± 7.123 | 64.819 ± 7.181 | 64.327 ± 6.884 | 0.509 |
| LOS in ICU, hours |  | 39.600 ± 12.572 | 39.811 ± 12.598 | 38.802 ± 12.442 | 0.443 |
| IABP or ECMO, n (%) |  | 1 (0.180) | 1 (0.228) | 0 (0.000) | 0.607 |
| Cardioversion, n (%) |  | 11 (1.982) | 8 (1.822) | 3 (2.586) | 0.600 |
| Permanent pacemaker, n (%) |  | 1 (0.180) | 1 (0.228) | 0 (0.000) | 0.607 |
| Hemodialysis, n (%) |  | 1 (0.180) | 1 (0.228) | 0 (0.000) | 0.607 |
| Postoperative AKI, n (%) |  | 1 (0.180) | 1 (0.228) | 0 (0.000) | 0.607 |
| Cerebral infarction, n (%) |  | 1 (0.180) | 1 (0.228) | 0 (0.000) | 0.607 |
| Length of hospital stay after surgery, days |  | 11.000 [8.000,16.000] | 11.000 [8.000,15.000] | 13.000 [9.000,18.000] | 0.007 |
| Follow-up time, years |  | 5.000 [3.000,6.000] | 4.000 [3.000,6.000] | 5.000 [3.000,6.000] | 0.222 |

AF, atrial fibrillation; LOS, length of stay; SBP, systolic blood pressure; DBP, diastolic blood pressure; BMI, body mass index; LAD, left atrial diameter; RAD, right atrial diameter; LVD, left ventricle diameter; RVD, right ventricle diameter; LVEF, left ventricular ejection fraction; CHD, coronary heart disease; PHTN, pulmonary hypertension; WBC, white blood cell count; NEUT%, neutrophil ratio; NLR, neutrophil-to-lymphocyte ratio; PLT, platelet; INR, international normalized ratio; PT, prothrombin time; AST, aspartate aminotransferase; ALT, alanine aminotransferase; Cr, Creatinine; BUN, Blood urea nitrogen; Tbil, total bilirubin; NYHA, New York Heart Association classification; AVR, aortic valve replacement; MVR, mitral valve replacement; MVP, mitral valvuloplasty; TVP, tricuspid valvuloplasty; CBP, cardiopulmonary bypass; AKI, acute kidney injury.

# Supplementary Table 2: Baseline, hospitalization and follow-up characteristics of the patients in the Non-CMP-IV group.

| n |  | Total (n=471) | AF non-recurrence (n=69) | AF recurrence (n=402) | P |
| --- | --- | --- | --- | --- | --- |
| Gender, n (%) |  | 343 (72.824) | 49 (70.000) | 294 (73.317) | 0.565 |
| Age, years |  | 57.416 ± 7.838 | 56.286 ± 7.459 | 57.613 ± 7.886 | 0.192 |
| Duration of AF, years |  | 3.000 [2.000,6.000] | 3.000 [2.000,5.000] | 3.000 [2.000,6.000] | 0.059 |
| Height, cm |  | 158.204 ± 7.275 | 157.800 ± 7.477 | 158.274 ± 7.237 | 0.616 |
| Weight, cm |  | 58.031 ± 9.525 | 58.853 ± 8.794 | 57.888 ± 9.640 | 0.435 |
| SBP, mmHg |  | 113.327 ± 14.478 | 114.000 ± 13.840 | 113.209 ± 14.583 | 0.674 |
| DBP, mmHg |  | 71.130 ± 13.257 | 70.471 ± 16.760 | 71.244 ± 12.542 | 0.715 |
| BMI, kg/m^2^ |  | 23.167 ± 3.362 | 23.650 ± 3.175 | 23.083 ± 3.387 | 0.193 |
| Preoperative heart rate, bpm |  | 87.100 ± 21.463 | 87.114 ± 23.549 | 87.097 ± 21.077 | 0.995 |
| Preoperative LAD, mm |  | 53.336 ± 10.894 | 48.971 ± 9.841 | 54.098 ± 10.890 | <0.001 |
| Preoperative RAD, mm |  | 40.098 ± 8.862 | 38.076 ± 7.779 | 40.451 ± 8.991 | 0.039 |
| Preoperative LVD, mm |  | 51.059 ± 8.758 | 51.383 ± 8.596 | 51.003 ± 8.784 | 0.738 |
| Preoperative RVD, mm |  | 38.008 ± 7.424 | 37.279 ± 8.461 | 38.135 ± 7.221 | 0.374 |
| Preoperative LVEF, % |  | 61.943 ± 8.888 | 61.571 ± 9.730 | 62.008 ± 8.731 | 0.705 |
| Preoperative hypertension,n (%) |  | 38 (8.068) | 7 (10.000) | 31 (7.731) | 0.52 |
| Preoperative diabetes, n (%) |  | 17 (3.609) | 1 (1.429) | 16 (3.990) | 0.289 |
| Preoperative CHD, n (%) |  | 31 (6.582) | 4 (5.714) | 27 (6.733) | 0.751 |
| History of preoperative cerebral infarction, n (%) |  | 23 (4.883) | 8 (11.429) | 15 (3.741) | 0.006 |
| Preoperative pulmonary hypertension, n (%) |  | 118 (25.053) | 18 (25.714) | 100 (24.938) | 0.89 |
| Smoking or drinking, n (%) |  | 19（4.034） | 4 (5.714) | 15 (3.741) | 0.439 |
| HAS-BLED Score, n (%) | 0 | 346 (73.461) | 46 (65.714) | 300 (74.813) | 0.085 |
|  | 1 | 106 (22.505) | 22 (31.429) | 84 (20.948) |  |
|  | 2 | 17 (3.609) | 1 (1.429) | 16 (3.990) |  |
|  | 4 | 2 (0.425) | 1 (1.429) | 1 (0.249) |  |
| CHA2DS2-VASc Score, n (%) | 0 | 120 (25.478) | 19 (27.143) | 101 (25.187) | 0.829 |
|  | 1 | 285 (60.510) | 41 (58.571) | 244 (60.848) |  |
|  | 2 | 32 (6.794) | 5 (7.143) | 27 (6.733) |  |
|  | 3 | 31 (6.582) | 4 (5.714) | 27 (6.733) |  |
|  | 4 | 1 (0.212) | 0 (0.000) | 1 (0.249) |  |
|  | 5 | 2 (0.425) | 1 (1.429) | 1 (0.249) |  |
| Preoperative NYHA, n (%) | I ＆II | 33 (7.006) | 4 (5.714) | 29 (7.232) | 0.719 |
|  | III | 366 (77.707) | 57 (81.429) | 309 (77.057) |  |
|  | IV | 72 (15.287) | 9 (12.857) | 63 (15.711) |  |
| Euro Score Ⅱ score |  | 1.650 ± 0.949 | 1.511 ± 0.678 | 1.674 ± 0.986 | 0.186 |
| Preoperative WBC, 10^12^/L |  | 5.936 ± 1.876 | 6.369 ± 2.326 | 5.861 ± 1.775 | 0.037 |
| Preoperative NEUT%, % |  | 58.595 ± 10.279 | 60.277 ± 10.177 | 58.301 ± 10.268 | 0.138 |
| Preoperative RBC, 10^12^/L |  | 4.783 ± 1.451 | 4.581 ± 1.194 | 4.819 ± 1.489 | 0.144 |
| Preoperative hemoglobin, g/L |  | 127.444 ± 15.622 | 128.057 ± 15.484 | 127.337 ± 15.643 | 0.722 |
| Preoperative neutrophil, 10^9^/L |  | 4.430 ± 1.771 | 4.475 ± 1.525 | 4.422 ± 1.810 | 0.82 |
| Preoperative lymphocyte, 10^9^/L |  | 1.858 ± 0.833 | 1.836 ± 0.737 | 1.862 ± 0.848 | 0.812 |
| Preoperative NLR |  | 2.355 [1.604,3.562] | 2.253 [1.790,3.317] | 2.372 [1.581,3.652] | 0.322 |
| Preoperative PLT, 10^9^/L |  | 186.463 ± 76.597 | 174.343 ± 68.133 | 188.579 ± 77.787 | 0.152 |
| Preoperative INR |  | 1.293 ± 1.324 | 1.169 ± 0.472 | 1.314 ± 1.421 | 0.399 |
| Preoperative PT, s |  | 13.274 ± 5.124 | 13.227 ± 5.149 | 13.282 ± 5.119 | 0.934 |
| Preoperative AST, U/L |  | 28.200 [21.100,40.700] | 27.700 [21.600,32.410] | 28.200 [21.000,43.890] | 0.393 |
| Preoperative ALT, U/L |  | 22.300 [15.700,38.100] | 19.600 [14.500,28.000] | 22.500 [15.900,38.900] | 0.083 |
| Preoperative Creatinine, µmol/l |  | 5.940 [4.450,7.800] | 5.810 [4.420,7.600] | 5.950 [4.450,7.910] | 0.509 |
| Preoperative BUN, mmol/L |  | 6.213 ± 2.503 | 5.954 ± 2.218 | 6.258 ± 2.547 | 0.348 |
| Preoperative Tbil, μmol/L |  | 15.700 [11.300,22.700] | 15.700 [12.200,22.800] | 15.600 [11.100,22.900] | 0.595 |
| Type of Surgery, n (%) | AVR | 8 (1.699) | 1 (1.429) | 7 (1.746) | 0.786 |
|  | DVR | 157 (33.333) | 24 (34.286) | 133 (33.167) |  |
|  | MVR | 272 (57.749) | 41 (58.571) | 231 (57.606) |  |
|  | TVP only | 7 (1.486) | 0 (0.000) | 7 (1.746) |  |
|  | MVP only | 19 (4.034) | 4 (5.714) | 15 (3.741) |  |
|  | TVR only | 8 (1.699) | 0 (0.000) | 8 (1.995) |  |
| MVP combined, n (%) |  | 19 (4.034) | 4 (5.714) | 15 (3.741) | 0.439 |
| TVP combined, n (%) |  | 273 (57.962) | 38 (54.286) | 235 (58.603) | 0.5 |
| TVR combined, n (%) |  | 27 (5.732) | 4 (5.714) | 23 (5.736) | 0.994 |
| CBP time, min |  | 102.737 ± 43.122 | 100.071 ± 42.934 | 103.202 ± 43.137 | 0.576 |
| Aortic obstruction time, min |  | 63.794 ± 31.113 | 64.014 ± 32.496 | 63.756 ± 30.865 | 0.949 |
| Left atrial thrombus, n (%) |  | 67 (14.225) | 8 (11.429) | 59 (14.713) | 0.468 |
| Postoperative heart rhythm, n (%) | Non-sinus rhythm | 262 (55.626) | 28 (40.000) | 234 (58.354) | 0.004 |
| Postoperative heart rate, bpm |  | 83.531 ± 21.436 | 82.286 ± 22.213 | 83.748 ± 21.289 | 0.599 |
| Postoperative LAD, mm |  | 44.128 ± 9.558 | 42.300 ± 8.609 | 44.447 ± 9.679 | 0.083 |
| Postoperative RAD, mm |  | 36.538 ± 5.046 | 35.313 ± 4.710 | 36.752 ± 5.072 | 0.028 |
| Postoperative LVD, mm |  | 47.128 ± 6.111 | 47.260 ± 6.918 | 47.105 ± 5.959 | 0.846 |
| Postoperative RVD, mm |  | 34.984 ± 5.063 | 34.033 ± 4.870 | 35.150 ± 5.078 | 0.089 |
| Postoperative LVEF, % |  | 64.831 ± 7.248 | 63.037 ± 8.911 | 65.144 ± 6.870 | 0.065 |
| LOS in ICU, hours |  | 39.259 ± 12.436 | 39.229 ± 10.860 | 39.264 ± 12.691 | 0.98 |
| IABP or ECMO, n (%) |  | 1 (0.212) | 0 (0.000) | 1 (0.249) | <0.001 |
| Cardioversion, n (%) |  | 19 (4.034) | 4 (5.714) | 15 (3.741) | 0.439 |
| Permanent pacemaker, n (%) |  | 6 (1.274) | 1 (1.429) | 5 (1.247) | 0.9 |
| Hemodialysis, n (%) |  | 1 (0.212) | 0 (0.000) | 1 (0.249) | <0.001 |
| Postoperative AKI, n (%) |  | 3 (0.637) | 1 (1.429) | 2 (0.499) | 0.367 |
| Postoperative Cerebral infarction, n (%) |  | 2 (0.425) | 1 (1.429) | 1 (0.249) | 0.162 |
| Length of hospital stay after surgery, days |  | 11.000 [8.000,15.000] | 10.000 [8.000,13.000] | 12.000 [8.000,15.000] | 0.061 |
| Follow-up time, years |  | 5.000 [3.000,6.000] | 4.000 [3.000,6.000] | 5.000 [3.000,6.000] | 0.222 |

AF, atrial fibrillation; LOS, length of stay; SBP, systolic blood pressure; DBP, diastolic blood pressure; BMI, body mass index; LAD, left atrial diameter; RAD, right atrial diameter; LVD, left ventricle diameter; RVD, right ventricle diameter; LVEF, left ventricular ejection fraction; CHD, coronary heart disease; PHTN, pulmonary hypertension; WBC, white blood cell count; NEUT%, neutrophil ratio; NLR, neutrophil-to-lymphocyte ratio; PLT, platelet; INR, international normalized ratio; PT, prothrombin time; AST, aspartate aminotransferase; ALT, alanine aminotransferase; Cr, Creatinine; BUN, Blood urea nitrogen; Tbil, total bilirubin; NYHA, New York Heart Association classification; AVR, aortic valve replacement; MVR, mitral valve replacement; MVP, mitral valvuloplasty; TVP, tricuspid valvuloplasty; TVR, tricuspid valve replacement; CBP, cardiopulmonary bypass; AKI, acute kidney injury.

# Methods

Five-fold cross-validation is a commonly used method for evaluating the performance of a model. It involves dividing the data into five mutually exclusive subsets and conducting five iterations where in each iteration, four folds are used for training and the remaining fold is used for validation. The average of the five evaluations is then calculated as the final model evaluation result. This method is useful for making better use of all the samples in the dataset and avoiding issues with inaccurate model evaluations due to poor dataset partitioning.

We calculated the feature importance scores for our four models, XGBoost, Catboost, Random Forest and LightGBM, using the default implementation of each algorithm. XGBoost utilized the Weight feature importance method, Catboost utilized the PredictionValuesChange method, LightGBM utilized the Gain method, and Random Forest utilized the Gini Importance method to calculate feature importance. We apologize for any confusion and thank you again for your valuable input.

# Introduction to the 10 Machine Learning Models

Support Vector Machine (SVM) is a type of supervised learning algorithm that can be used for classification or regression tasks. They work by finding the hyperplane in a high-dimensional space that maximally separates the different classes.

Logistic Regression is a type of regression analysis that is used to predict the probability of an event occurring. It works by using an equation to predict the probability that an event will occur based on one or more independent variables.

XGBoost is an open-source software library that provides an efficient implementation of gradient boosting for machine learning. It is designed to be highly efficient, flexible, and portable.

Random Forest is a type of ensemble learning method for classification, regression, and other tasks, that operate by constructing a multitude of decision trees at training time and outputting the class that is the mode of the classes (classification) or mean prediction (regression) of the individual trees.

CatBoost is a gradient boosting library developed by Yandex. It is designed to improve the performance of gradient boosting algorithms on categorical data.

AdaBoost (Adaptive Boosting) is an ensemble learning algorithm that can be used for classification or regression. It works by weighting the instances in the training set based on their difficulty to classify, and subsequently training a classifier on the weighted training set.

Bagging (Bootstrapped Aggregation) is an ensemble method that involves training multiple models on different random subsets of the training data and then averaging their predictions. It is often used to reduce the variance of a model.

Gradient Boosting Decision Trees (GBDT) is a type of boosting algorithm that uses decision trees as the weak learner. It works by sequentially training decision trees and then combining them to make a final prediction.

LightGBM is a gradient boosting library developed by Microsoft that is designed to be efficient and scalable. It is particularly well-suited for working with large-scale data sets.

Multilayer Perceptron (MLP) is a type of feedforward artificial neural network that is composed of multiple layers of interconnected nodes. It is often used for classification and regression tasks.

# Supplementary Table 3: Missing values and dispositions

| Variables | Total | No of missing values (Non-CMP-IV group) | No of missing values (CMP-IV group) |
| --- | --- | --- | --- |
| Duration of AF | 5 (0.49%) | 3 (0.64%) | 2 (0.36%) |
| SBP | 11 (1.07%) | 8 (1.70%) | 3 (0.54%) |
| DBP | 11 (1.07%) | 8 (1.70%) | 3 (0.54%) |
| Preoperative heart rate | 10 (0.97%) | 6 (1.27%) | 4 (0.72%) |
| Preoperative NYHA | 12 (1.17%) | 5 (1.06%) | 7 (1.26%) |
| Preoperative WBC | 15 (1.46%) | 9 (1.91%) | 6 (1.08%) |
| Preoperative NEUT% | 15 (1.46%) | 9 (1.91%) | 6 (1.08%) |
| Preoperative RBC | 15 (1.46%) | 9 (1.91%) | 6 (1.08%) |
| Preoperative hemoglobin | 15 (1.46%) | 9 (1.91%) | 6 (1.08%) |
| Preoperative neutrophil | 15 (1.46%) | 9 (1.91%) | 6 (1.08%) |
| Preoperative lymphocyte | 15 (1.46%) | 9 (1.91%) | 6 (1.08%) |
| Preoperative PLT | 15 (1.46%) | 9 (1.91%) | 6 (1.08%) |
| Preoperative INR | 20 (1.95%) | 11 (2.34%) | 9 (1.62%) |
| Preoperative PT | 20 (1.95%) | 11 (2.34%) | 9 (1.62%) |
| Preoperative AST | 12 (1.17%) | 7 (1.49%) | 5 (0.90%) |
| Preoperative ALT | 12 (1.17%) | 7 (1.49%) | 5 (0.90%) |
| Preoperative Creatinine | 12 (1.17%) | 7 (1.49%) | 5 (0.90%) |
| Preoperative BUN | 12 (1.17%) | 7 (1.49%) | 5 (0.90%) |
| Preoperative Tbil | 12 (1.17%) | 7 (1.49%) | 5 (0.90%) |

AF, atrial fibrillation; SBP, systolic blood pressure; DBP, diastolic blood pressure; NYHA, New York Heart Association classification; WBC, white blood cell count; NEUT%, neutrophil ratio; PLT, platelet; INR, international normalized ratio; PT, prothrombin time; AST, aspartate aminotransferase; ALT, alanine aminotransferase; Cr, Creatinine; BUN, Blood urea nitrogen; Tbil, total bilirubin
